# Dietary polyphenols and human health: sources, biological activities, nutritional and immunological aspects, and bioavailability– a comprehensive review

**DOI:** 10.3389/fimmu.2025.1653378

**Published:** 2025-11-03

**Authors:** Ahmed M. Saad, Dina Mostafa Mohammed, Samar Sami Alkafaas, Soumya Ghosh, Shaimaa H. Negm, Heba M. Salem, Mohamed A. Fahmy, Hatem E. Semary, Essam H. Ibrahim, Synan F. AbuQamar, Khaled A. El-Tarabily, Mohamed T. El-Saadony

**Affiliations:** ^1^ Department of Biochemistry, Faculty of Agriculture, Zagazig University, Zagazig, Egypt; ^2^ Nutrition and Food Sciences Department, National Research Centre, Giza, Egypt; ^3^ Molecular Cell Biology Unit, Division of Biochemistry, Department of Chemistry, Faculty of Science, Tanta University, Tanta, Egypt; ^4^ Natural and Medical Sciences Research Center, University of Nizwa, Nizwa, Oman; ^5^ Department of Genetics, University of the Free State, Bloemfontein, South Africa; ^6^ Department of Home Economics, Specific Education Faculty, Port Said University, Port Said, Egypt; ^7^ Department of Poultry Diseases, Faculty of Veterinary Medicine, Cairo University, Giza, Egypt; ^8^ Department of Agricultural Microbiology, Faculty of Agriculture, Zagazig University, Zagazig, Egypt; ^9^ Department of Mathematics and Statistics, Faculty of Science, Imam Mohammed Ibn Saud Islamic University (IMSIU), Riyadh, Saudi Arabia; ^10^ Biology Department, Faculty of Science, King Khalid University, Abha, Saudi Arabia; ^11^ Department of Biology, College of Science, United Arab Emirates University, Al Ain, United Arab Emirates

**Keywords:** antioxidant, bioavailability, biological properties, immunological properties, polyphenols

## Abstract

Dietary polyphenols, particularly flavonoids, have been extensively recognized for their role as a source of bioactive molecules that contribute to the prevention of various diseases, including cancer. This review aims to provide a comprehensive overview of dietary polyphenols by examining their sources, classification, mechanisms of action, and biological effects, with a particular emphasis on their nutritional and immunological roles. It also highlights the need for ongoing research into preventive strategies and the development of improved therapeutic options. Despite their broad spectrum of antioxidant, anti-inflammatory, neuroprotective, antimicrobial, anti-diabetic, and anti-cancer activities, the therapeutic application of polyphenols is significantly hindered by their inherently poor bioavailability. This limitation poses a substantial challenge, as it prevents polyphenols from achieving the systemic concentration necessary to elicit a therapeutic effect. This review critically evaluates current strategies, including nano- and liposomal-based delivery systems. Liposomal systems play a crucial role in enhancing the bioavailability of polyphenols by encapsulating these compounds in lipid bilayers. This encapsulation improves the solubility and stability of polyphenols, protects them from environmental degradation and rapid metabolism, and facilitates their controlled release and absorption in the body. Liposomes enable polyphenols to better traverse biological membranes and protect them from unfavorable conditions in the gastrointestinal tract, resulting in greater systemic availability and improved therapeutic efficacy compared to non-encapsulated forms. The current review also explores the modulatory impact of polyphenols on the immune system, their influence on gut microbiota, and their implications across various life stages, from infancy to aging, as well as in athletic performance and dermatological health. Future directions are proposed to optimize their clinical utility, including standardized dosing, improved delivery technologies, and targeted nutritional interventions. Ultimately, integrating polyphenols into daily dietary practices may offer promising avenues for enhancing immune resilience and preventing chronic diseases.

## 1 Introduction

Life expectancy in developing nations is increasing in tandem with socioeconomic progress. As a result of this shifting lifestyle, age-related illnesses, such as cancer, diabetes, cardiovascular disease, metabolic disorders, hepatitis, and neurological conditions, are on the rise (1). The absence of early detection technologies or effective treatments has prompted researchers to focus on preventive measures (1). In this context, attention has turned to dietary and nutritional strategies, such as the Mediterranean diet. These dietary habits may mitigate the risk of age-related disorders associated with lifestyle changes (1). Predominantly based on plant-derived foods such as vegetables, fruits, legumes, and herbs, the Mediterranean diet highlights the potential role of natural polyphenols, plant-based bioactive compounds, in preventing disease and aging while promoting overall health and well-being (2).

Polyphenols are naturally occurring, water-soluble compounds derived from plants, with molecular weights ranging from 500 to 4000 Da. They are abundant in plant-based foods, including fruits, vegetables, cereals, and beverages, and comprise a complex group of over 8000 known compounds (3). These compounds are classified as secondary metabolites (4), which are produced to defend against biotic stressors (e.g., bacteria, fungi, and insects) and abiotic stressors (e.g., environmental stress, free radicals, and metabolic disorders) (5, 6).

Based on the number of phenolic rings and structural linkages, they are commonly categorized into five main classes: tannins, lignans, phenolic acids, flavonoids, and stilbenes (7). They exhibit a wide range of biological activities, including anti-inflammatory, anti-cancer, antimicrobial, and anti-aging effects, due to their structural properties and biological interactions (8, 9). Consequently, they have shown great potential in the management of various diseases, including cancers and neurological, cardiovascular, and metabolic conditions (8, 10).

This review highlights the major classes of polyphenols, evaluated as secondary metabolites, along with methods used for their extraction and characterization. It also outlines their bioavailability and diverse health benefits, as reported in previous studies. In addition, the advantages of polyphenol consumption across different population groups, including athletes, mothers, infants, children, adults, and the elderly, are discussed.

## 2 Types of polyphenols

The basic phenolic structure of polyphenols is exemplified by these naturally occurring compounds, which are classified according to their chemical composition, particularly the number of aromatic rings, the substituent groups on these rings, and the structural linkages between them (7).

**Figure 1:**
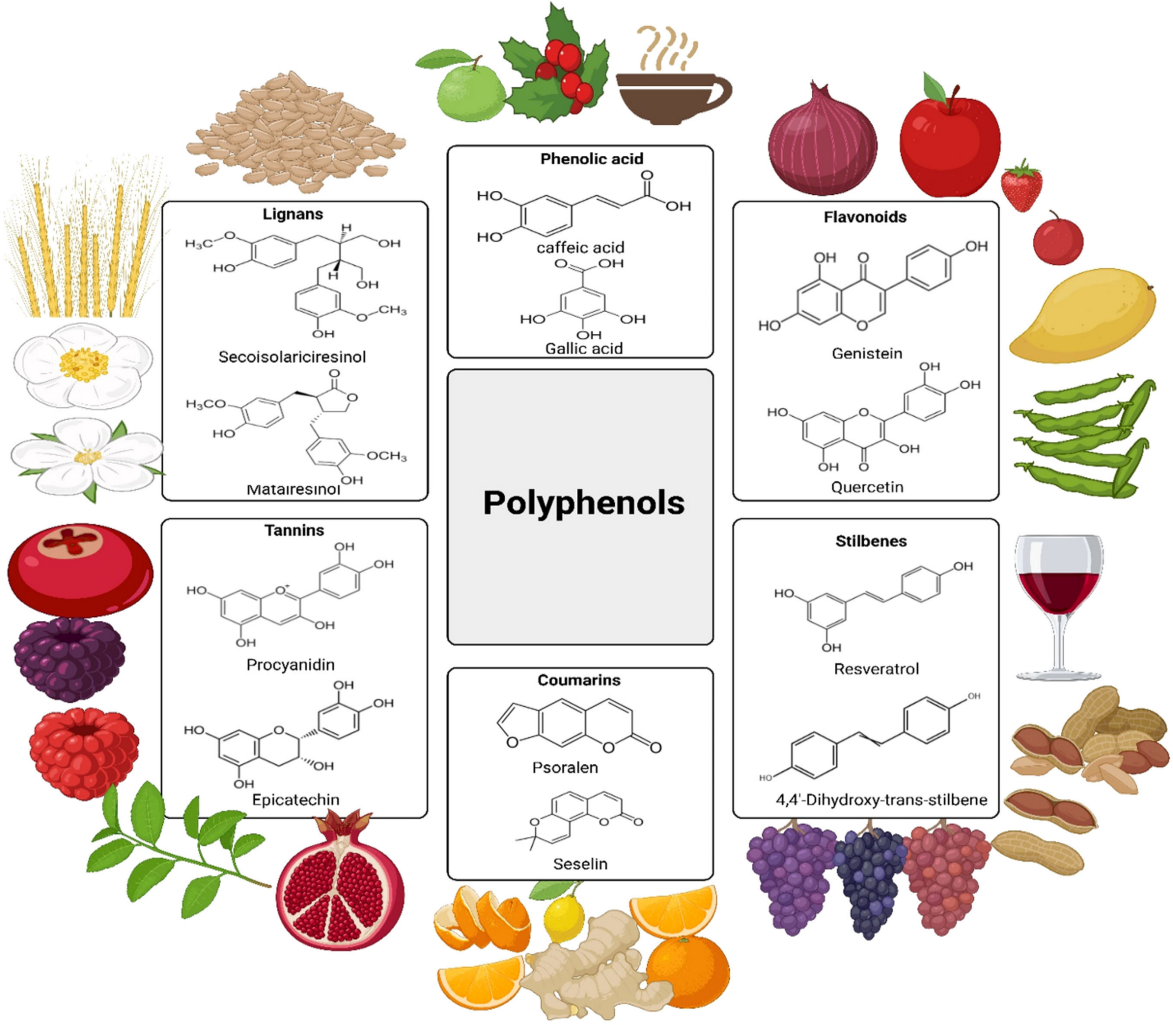
Chemical structures of principal polyphenol subclasses (lignans, phenolic acids, flavonoids, tannins, coumarins, and stilbenes) alongside their respective plant-based food sources exemplifying dietary consumption.

**Figure 1** depicts the chemical structures of essential polyphenol subclasses (lignans, phenolic acids, flavonoids, tannins, coumarins, and stilbenes) along with their respective plant-based food sources, highlighting dietary consumption sources.

### 2.1 Phenolic acids and flavonoids

Phenolic acids differ from other acids in that they contain a single phenolic ring, characterized by the presence of one carboxylic acid group and one or more hydroxyl groups (11). Phenolic acids are structurally similar to other phenolic compounds. As a result, phenolic acids are commonly associated with esters, amides, and glycosides (11).

They are generally divided into two major subgroups: hydroxybenzoic acids and hydroxycinnamic acids. Hydroxybenzoic acid, derived from benzoic acid, possesses a C_6_-C_1_ carbon structure, while hydroxycinnamic acids, derived from cinnamic acid, typically occur in plants as simple esters with quinic acid or glucose (12). Phenolic acids are widely distributed in various foods, particularly cereals, fruits, legumes, vegetables, herbs, and beverages (13).

Flavonoids are the most well-known and extensively studied class of polyphenols (13). Their basic structure consists of two aromatic rings connected by a three-carbon bridge, forming an oxygen-containing heterocyclic ring. Based on the degree of oxidation of the central carbon ring, flavonoids are categorized into six major subclasses: flavonols, flavanones, flavones, flavanols, isoflavones, and anthocyanidins (13, 14). Flavonoids, identified as secondary metabolites in specific plant structures such as seeds and fruits, play a crucial role in contributing to the color, flavor, and aroma of plants. This structurally diverse group of polyphenols that exists in various forms is among the most thoroughly investigated in plant science (13).

Phenolic acids could donate hydrogen atoms, suggesting their antioxidant properties (13). Additionally, they are notable for their therapeutic properties in managing several chronic conditions, including diabetes, cardiovascular diseases, cancer, and neurodegenerative conditions (15, 16). Their fundamental structural features, the aromatic rings, hydroxyl groups (-OH) at specific positions, and the unsaturated side chains, contribute to a range of biological activities, with particular emphasis on anti-cancer effects (17).

On the other hand, flavonoids contribute to activating defense responses by modulating the production of reactive oxygen species (ROS) under stress conditions (18, 19). Accordingly, flavonoids demonstrate a wide range of bioactive properties beneficial to human health, including anti-inflammatory, antioxidant, cardioprotective, neuroprotective, anti-cancer, and anti-aging effects (20, 21).

### 2.2 Stilbenes

This class of polyphenols represents a distinct group of non-flavonoid phytochemicals characterized by two aromatic rings linked by a methylene bridge (13). Stilbenes are structurally defined by a 1,2-diphenylethylene core, distinguishing them as a subclass of phenylpropanoids. Resveratrol, the most prominent stilbene, is naturally found in peanuts and grapes and is present in high concentrations in red wine (14, 22).

The abundance of resveratrol in red wine has contributed to hypotheses regarding its potential role in preventing chronic diseases (23). Additionally, resveratrol has been reported to possess anti-inflammatory and antioxidant properties (14, 24). It has also been suggested to contribute to wine preservation (25). Previous studies reported the development of a wine quality index based on the concentration and composition of stilbenes (26).

### 2.3 Lignans

Lignans are another class of polyphenols that share structural similarities with phenolic acids (14). Their diphenolic structure includes a carbon-carbon bond formed between two phenylpropane units, which polymerize to produce compounds commonly found in plant seeds, roots, and leaves (27). Lignans are classified into eight subgroups based on their cyclization pattern, the incorporation of oxygen atoms, and carbon skeleton structures. These subclasses include furofurans, furans, aryltetralins, arylnaphthalenes, dibenzylbutyrolactones, dibenzylbutanes, dibenzybutyrolactols, and dibenzocyclooctadienes (27). Li et al. (27) reported that the position of the oxygen atom also plays a key role in the classification of lignans.

Lignans are primarily found in vegetables, cereals, and legumes. Diets rich in lignans have been associated with various health-promoting effects. Notably, lignans exhibit anti-cancer activity through multiple regulatory pathways (28, 29). Furthermore, they possess anti-inflammatory, antioxidant, and anti-menopausal properties, providing protective effects against cardiovascular and bone diseases, as well as antimicrobial effects (30, 31).

## 3 Factors affecting the appropriate extraction methods for phenolic compounds

Polyphenols comprise a broad array of chemical structures, resulting in varied chemical and physical properties (32). This structural heterogeneity necessitates the application of extraction techniques specifically designed for the distinct properties of each compound and the nature of the sample matrix (32). The selection of an efficient extraction method depends on various aspects, including the chemical structure of the target polyphenols, the sample’s particle size, and the existence of other coexisting chemicals that may interfere or interact during the extraction process (33).

Furthermore, extraction efficiency is highly sensitive to operational parameters such as pH, type of solvent employed, solvent-to-sample ratio, and duration of the extraction process (34). Despite considerable technological progress in extraction techniques, significant challenges remain in precisely identifying and quantifying polyphenols. Obtaining accurate and reproducible data on the composition and concentration of polyphenols is critical for substantiating their health-promoting properties and ensuring the reliability of related scientific assessments (35).

### 3.1 Extraction methods of phenolic compounds

#### 3.1.1 Ultrasound-assisted extraction method

Ultrasound-assisted extraction is a widely used and efficient technique for isolating phenolic compounds, offering high yields in a relatively short time (36). Ultrasonic radiation with frequencies above 20 kHz enhances the extraction of inorganic and organic substances using liquid solvents. This method is considered environmentally sustainable as it reduces extraction time, solvent consumption, and energy requirements (37).

The process relies on acoustic cavitation, wherein ultrasonic waves disrupt plant cell walls by inducing rapid expansion and contraction of solid surfaces (38), increasing cell wall permeability, facilitating solvent penetration, and promoting the release of water-soluble compounds from the plant matrix (37). In recent years, ultrasound-assisted extraction has been applied to extract polyphenols from various plant parts, including pecan nutshells, *Randia monantha*, mango seed kernels, olive pomace, and pine needles (36, 39). Studies have focused on optimizing extraction conditions and evaluating the antioxidant and antifungal properties of the resulting polyphenol-rich extracts (36, 37, 39).

Ultrasound-assisted extraction provides notable advantages for the extraction of polyphenols. This technique significantly enhances yield and efficiency compared to conventional methods, allowing for higher concentrations of bioactive compounds in a much shorter extraction time (40). Ultrasound-assisted extraction reduces both solvent and energy consumption, making the process more environmentally friendly and cost-effective. The operation at lower temperatures also helps preserve the structural integrity of heat-sensitive polyphenols, minimizing their thermal degradation during extraction (40). These benefits are reflected in various studies, which demonstrate that the ultrasound-assisted extraction delivers superior extraction performance while aligning with green chemistry principles (40).

However, the ultrasound-assisted extraction also has its limitations (41). Excessive ultrasound intensity or prolonged application can lead to the generation of free radicals and high local temperatures, potentially causing the degradation or modification of certain sensitive polyphenolic compounds (41). The method’s efficacy is highly dependent on the careful optimization of operating parameters, such as ultrasound power, extraction time, solvent type, and temperature, as suboptimal conditions may reduce extraction yields or lead to inconsistent results (41). Additionally, scaling up the ultrasound-assisted extraction from laboratory to industrial production remains challenging due to equipment limitations and the need to replicate the cavitation effects that drive the process consistently (41).

#### 3.1.2 Microwave-assisted extraction method

Microwave-assisted extraction is an environmentally friendly technique used to isolate polyphenols from plants, herbs, and plant-based products (42). Water is commonly used as the solvent due to its efficiency, cost-effectiveness, and reliability compared to other extraction media. Optimizing operational parameters is essential, as the heat generated during microwave exposure influences the release of targeted polyphenols (42). Extraction efficiency depends on factors such as solvent type and ratio, microwave power, and extraction time (43). These conditions must be maintained and optimized to obtain the highest yield. Depending on the plant sample, the solvent can be water, ethanol, or a combination (43).

Microwave-assisted extraction is frequently employed to recover polyphenols from agricultural by-products and processed wastes, such as pomace, leaves, and peels (44). Extracted polyphenols by the microwave-assisted extraction method have demonstrated various biological activities, including antimicrobial, antioxidant, and anti-cancer activities (44, 45). These bioactive compounds have potential applications in pharmaceuticals and nutraceuticals (43, 46, 47).

This technique significantly reduces extraction time and energy consumption compared to conventional methods. Microwave-assisted extraction enhances extraction efficiency by rapidly heating the sample and solvent, which disrupts plant cell walls and allows better solvent penetration, leading to higher yields of polyphenolic compounds (41). The method also offers improved selectivity and precise control of temperature, helping preserve the integrity of thermosensitive compounds (41).

Nonetheless, microwave-assisted extraction possesses limitations. Excessive microwave power or prolonged exposure can lead to the degradation of heat-sensitive or volatile phenolics, thus reducing the quality and quantity of the extracted compounds (48). Additionally, optimization is required for each plant matrix and polyphenol type, as extraction parameters such as microwave power, temperature, solvent composition, and sample-to-solvent ratio can significantly influence outcomes (48). Some specialized equipment and careful calibration are needed to ensure reproducibility and scalability for industrial applications (48).

#### 3.1.3 Microwave-assisted ultrasound extraction method

Microwave-assisted ultrasound extraction presents notable advantages for extracting polyphenols (49). Microwave-assisted ultrasound extraction is a hybrid method that combines microwave and ultrasonic treatments to enhance the yield of phenolic compounds, reduce extraction time, and minimize solvent usage compared to ultrasound-assisted extraction and microwave-assisted extraction alone (49). This method employs microwave heating to extract compounds through dielectric heating, while ultrasound enhances cell wall permeability and facilitates solvent penetration. A comparative study reported that microwave-assisted extraction yielded higher polyphenol content and antioxidant activity than ultrasound-assisted extraction (50).

While microwave-assisted extraction required less extraction time, ultrasound-assisted extraction demonstrated greater energy efficiency and environmental sustainability (51). When both techniques were combined, the resulting method improved extraction efficiency and polyphenol yield. For instance, a study comparing enzyme-assisted ultrasound extraction and ultrasound-microwave-assisted extraction from mangosteen peels found that the enzyme-assisted ultrasound method produced a higher polyphenol yield (51). Nonetheless, both extraction approaches yielded polyphenol-rich extracts with promising applications as functional food additives and in pharmaceutical formulations (51).

By combining microwave and ultrasound energies, microwave-assisted ultrasound extraction disrupts plant cell walls more efficiently, allowing improved release of polyphenols with reduced solvent consumption and lower energy usage (51). Additionally, the process can preserve the antioxidant activities of the extracted compounds and is considered both cost-effective and environmentally friendly (48).

However, there are some limitations. Precise control of operational parameters—such as power, temperature, and extraction duration—is essential, as excessive energy input or prolonged treatment can degrade sensitive polyphenolic structures, potentially lowering yield or altering compound profiles (52). Variability in sample characteristics and the risk of free radical generation during ultrasound application can also influence extraction efficiency and product quality (52). Despite these challenges, when carefully optimized, microwave-assisted ultrasound extraction remains a powerful, green technology for extracting high-value polyphenols from complex plant matrices (52).

#### 3.1.4 Supercritical fluid extraction method

Supercritical fluid extraction is an alternative two-step technique. First, soluble phenolic compounds are extracted from the herbal cell matrix using a supercritical fluid, followed by depressurization to separate the bioactive components, converting the supercritical fluid into a gas phase (53, 54). Supercritical fluids are generated when pressure (10–35 MPa) and temperature (40–80 °C) exceed critical values. This method enhances safety by using less hazardous solvents, such as methyl tert-butyl ether, methanol, hexane, and dichloromethane (55).

Supercritical fluid extraction is considered a green technology, frequently employing gases like CO_2_, CH_3_, C_2_H_6_, C_2_H_6_O, C_3_H_8_, C_6_H_6_, and NH_3_ during depressurization (56). Additionally, compared to conventional methods, supercritical fluid extraction protects bioactive compounds from air and light, reducing degradation and minimizing contamination risk from impure solvents (57). This technique has recently been applied to extract polyphenols from sources such as chestnut shells, *Ailanthus excelsa*, and *Dunaliella salina* (54, 55, 58).

Supercritical fluid extraction, particularly with supercritical carbon dioxide (CO_2_), offers several advantages for the extraction of polyphenols (59). It is an environmentally friendly “green” technology that uses non-toxic, non-flammable CO_2_, resulting in solvent-free extracts that are safe for food, pharmaceutical, and cosmetic uses. This method operates at moderate temperatures, which helps preserve the structural integrity and bioactivity of heat-sensitive polyphenols (59). Supercritical fluid extraction also features tunable selectivity; by adjusting pressure, temperature, and the use of co-solvents such as ethanol or water, it can be optimized for higher purity and targeted extraction of diverse phenolic compounds (59). Additionally, supercritical fluid extraction minimizes solvent residue and maintains high extract quality, thus supporting the production of high-purity polyphenols (59).

However, supercritical fluid extraction also has limitations. The high initial cost and technical complexity of the required equipment are significant barriers to large-scale industrial application (60). Extraction efficiency can be lower for highly polar compounds unless co-solvents are used to enhance solubility (60). The method typically requires longer extraction times compared to some alternative techniques, and optimizing operational parameters such as pressure, temperature, and co-solvent composition can be challenging. Furthermore, scaling up the process for industrial throughput poses logistical and operational hurdles, and energy consumption is relatively high due to the need to maintain supercritical conditions (60).

#### 3.1.5 Subcritical water extraction method

Also known as hot liquid or superheated water extraction (61). In subcritical water extraction, water remains in a liquid state at temperatures between 100°C and 347°C under pressures up to 220 bar (62). Under these subcritical conditions, hydrogen bonding between water molecules is reduced, lowering the dielectric constant. Consequently, changes in temperature and pressure influence both the dielectric constant and extraction efficiency (62). Compared to supercritical fluid extraction, subcritical water extraction is potentially more economical as it utilizes water rather than organic solvents (63). Subcritical water extraction also produces rapid extraction, high efficiency, and environmental sustainability (64). It has been successfully applied to extract phenolic and natural compounds from materials such as cocoa bean husks and saffron tepals (62, 64, 65).

Subcritical water extraction offers green, efficient technology for polyphenol extraction, but has several limitations. A primary drawback of subcritical water extraction is the requirement for high temperatures, typically between 100°C and 374°C, which can cause thermal degradation of heat-sensitive polyphenolic compounds, thereby reducing their yield and bioactivity (66).

Moreover, at elevated temperatures, subcritical water extraction tends to be less selective, extracting a wider range of plant matrix components, which can complicate downstream purification (66). The use of water as a solvent under subcritical conditions also necessitates additional steps, such as evaporation or dehydration, to remove water from the extracts, increasing processing complexity (66).

Subcritical water extraction equipment requires more rigorous maintenance and corrosion prevention due to the high reactivity and corrosiveness of water at elevated temperature and pressure (66). Lastly, optimization of variables such as temperature, extraction time, pressure, and solvent-to-solid ratio is critical yet challenging, as these parameters profoundly influence extraction efficiency and compound stability (66). Thus, while subcritical water extraction is promising and eco-friendly, its limitations in compound stability, selectivity, and process complexity require careful management to maximize polyphenol recovery and bioactivity (66).

#### 3.1.6 Pulsed electric field method

A nonthermal method that employs high-voltage pulses between two electrodes arranged in a sandwich configuration. Pulsed electric field is classified into batch (100–300 V/cm) and continuous (20–80 kV/cm) systems, depending on pulse frequency. The electric field induces a transmembrane potential in plant cells, increasing membrane permeability and facilitating the excretion of phenolic compounds (67). Pulsed electric field effectiveness depends on the extent, the surrounding medium, and the physicochemical properties of plant tissues (68, 69). This method has been used to extract polyphenols from green tea, laurel leaves, cannabis, and *Phyllanthus emblica*, with extracts showing anti-inflammatory and antioxidant activity (70, 71).

Pulsed electric field technology, while promising as a non-thermal and efficient method for extracting polyphenols, has several limitations that should be considered. The effectiveness of pulsed electric field extraction depends on various factors, including the electric field strength, treatment time, and the specific properties of the plant tissue, such as cell size, shape, and membrane composition (72). One key limitation is the challenge of achieving a uniform electric field distribution throughout the sample, which can result in inconsistent cell permeabilization and variable extraction yields. Additionally, pulsed electric field treatment may cause only reversible electroporation in some cells, limiting the release of intracellular compounds (72).

Another constraint is related to the physical and chemical characteristics of the extraction matrix; factors such as solvent type, solvent conductivity, and polarity significantly influence extraction efficiency and can complicate optimization (73). Furthermore, pulsed electric field is typically better suited for liquid or semi-liquid matrices and may be less effective for solid or highly fibrous plant materials without prior size reduction or pretreatment (73). Although considered a non-thermal process, extended treatment times or high pulse numbers can lead to a rise in temperature, risking the degradation of sensitive phenolic compounds (73).

#### 3.1.7 Pressurized liquid extraction method

Also referred to as accelerated solvent extraction (74). It typically employs organic solvents in the presence of nitrogen to extract phenolic compounds from solid or semi-solid samples. Operating at high temperatures and pressures, accelerated solvent extraction enhances solvent penetration without altering compound structure, thereby improving phenolic yield (56). This green extraction method minimizes solvent and energy use while increasing extraction efficiency. Automation enhances process reproducibility with minimal manual intervention (75). Accelerated solvent extraction has been used to extract phenolics from strawberry and onion peels, with applications focused on evaluating their antimicrobial and antibiofilm activities (75, 76).

Pressurized liquid extraction offers efficient recovery of polyphenols from various plant matrices; however, it also presents certain limitations. One key challenge is the high operational cost associated with the specialized equipment and maintenance requirements (77). Additionally, pressurized liquid extraction involves the application of elevated temperatures and pressures, which can potentially lead to the degradation of thermolabile polyphenolic compounds, thereby reducing the yield and altering the composition of the extracts (77).

The choice of solvent is critical, as water, often used for its green credentials, may be inefficient in extracting less polar phenolics, resulting in lower overall extraction efficiency compared to organic solvents like ethanol (77). Optimization of operational parameters such as temperature, solvent composition, solvent-to-feed ratio, and extraction time is essential but can be complex and sample-specific, especially when dealing with complex matrices like propolis (77). Moreover, while pressurized liquid extraction reduces solvent use and extraction time compared to traditional methods, incomplete extraction of certain compounds can still occur, necessitating complementary techniques or further refinement (77). Finally, the process demands careful balancing between maximizing extraction efficiency and preventing compound degradation, which remains a key limitation in fully harnessing pressurized liquid extraction for polyphenol extraction (77).

### 3.2 Common methods for polyphenol quantification

#### 3.2.1 Spectrophotometric methods

Spectrophotometry is a simple and widely used technique for identifying phenolic compounds in plants (78). Total phenolic content is commonly assessed using the Folin–Denis and Folin–Ciocalteu methods. These techniques have recently been applied to evaluate the phenolic content, antioxidant activity, and total phenolics in broken-bone twigs (79, 80). Both methods rely on chemical reduction, typically involving reagents such as molybdenum and tungsten (81). Additionally, colorimetric assays are used to quantify total flavonoids, condensed tannins, and phenolics by forming complexes with AlCl_3_, with absorbance measured in the 410–423 nm range (82).

Anthocyanins, another important group of phenolics, can be quantified spectrophotometrically under mildly acidic conditions, with absorbance measured between 490 and 550 nm (83). These colorimetric assays are user-friendly and cost-effective; however, they do not allow for the quantification of individual compounds and provide only approximate estimates of total phenolics above a certain threshold (61). Despite this limitation, spectrophotometric methods remain valuable for the rapid and economical screening of a wide range of plant-derived bioactive compounds. For instance, red poppy extracts have recently been used as colorimetric sensors to detect anthocyanins (84), and similar analyses have been conducted on grape juice and elderberries (85, 86).

#### 3.2.2 Gas chromatography method

Gas chromatography is widely used to identify and quantify polyphenols, including flavonoids, phenolic acids, and tannins (87). This technique involves the movement of analytes through a column using carrier gas such as nitrogen (N_2_), helium (He), or hydrogen (H_2_). Gas chromatography operates based on gas-liquid partitioning or gas-solid adsorption, utilizing a nonvolatile liquid as the stationary phase and typically employing a flame ionization detector. Commonly, silica capillary columns are used, typically 30 m in length, with a 0.25 µm film thickness and an inner diameter of 25–32 µm (56).

The integration of gas chromatography with mass spectrometry (GC-MS) has gained attention owing to its improved sensitivity and selectivity (88). This combination is crucial for analyzing the degradation patterns of plant-derived bioactive compounds and for identifying their chemical structures by correlating chromatographic and mass spectral data (89). Gas chromatography analysis was used to evaluate the antimicrobial properties and polyphenol content of *Sonneratia caseolaris* fruits, as well as to determine the bioactive compound composition of fast-growing plant leaves (90).

#### 3.2.3 High-performance liquid chromatography method

High-performance liquid chromatography (HPLC) remains one of the most widely used analytical methods for the identification of phenolic compounds. Generally, following the purification of phenolics, the samples are analyzed using a C18 column as the stationary phase (91). This technique uses acidified polar organic solvents as the mobile phase and utilizes photodiode array detectors for compound detection. With technological advancements, rapid and refined methods such as chromatographic fingerprint analysis have been developed for the characterization of herbal medicines (56). These fingerprint profiles enable species-specific identification and differentiation from related species, as they accurately reflect the chemical composition of the plant material (56).

Several factors affect the sensitivity or effectiveness of HPLC, including phenolic purification steps, mobile phase composition, column selection, and pre-concentration procedures (35). The pH of the mobile phase is particularly critical, as improper pH levels may lead to the ionization of phenolic compounds, affecting detection accuracy (35). Column selection is based on polarity and particle size, with various phenolic classes requiring different specifications. More sophisticated HPLC systems employ novel column types with varying particle sizes to optimize separation (35).

HPLC run times typically range from 10 to 150 min. For longer analyses, maintaining a constant temperature is essential to ensure reproducibility and stability of results (92). Recent studies using HPLC have successfully characterized the antioxidant and antimicrobial properties, metabolomic profiles, and phenolic components of samples such as apple pomace, grape juice, *Lysimachia nummularia*, and *Acacia* species (93, 94).

#### 3.2.4 Other methods for polyphenol quantification

In addition to widely used techniques, several other methods are employed to identify plant-derived bioactive compounds, including capillary electrophoresis, paper chromatography, supercritical fluid chromatography, spectrophotometric assays, HPLC, and gas chromatography (78). Among these, paper chromatography is a simple and effective method, particularly for identifying bioactive compounds in tea leaves (78). Ashraf et al. (95) demonstrated the application of high-performance thin-layer chromatography in analyzing caffeine content in green tea leaves. Paper chromatography has also been applied to assess the biological activities of medicinal herbal extracts, such as anti-inflammatory, antimicrobial, and antioxidant properties linked to compounds like flavonoids and fatty acids (96).

However, paper chromatography is used less frequently than HPLC and gas chromatography due to its limited sensitivity and specificity (56). Capillary electrophoresis is a high-efficiency technique that utilizes thin capillary columns filled with ionic solutions to separate charged bioactive compounds and low-to-medium-molecular-weight plant constituents. It requires minimal sample and reagent volumes and offers rapid and effective analysis (56). Capillary electrophoresis techniques include micellar electrokinetic chromatography, capillary electrochromatography, capillary zone electrophoresis with ultraviolet detection, and capillary zone electrophoresis coupled with mass spectrometry (56). Recent applications of capillary electrophoresis include the quantification of free sulfur dioxide in wine and cider and the chemical profiling of tobacco samples (97, 98). Additionally, indirect UV detection with capillary zone electrophoresis has been used to investigate cassines and spectalines in *Senna spectabilis* (99).

Supercritical fluid chromatography is an advanced method increasingly used for the analysis of complex plant materials (100). Compared to HPLC and gas chromatography, supercritical fluid chromatography exhibits higher efficiency, faster analysis times, environmentally friendly operation, and superior resolution (56). Its distinguishing feature lies in column design, which incorporates fully porous particles smaller than 2 µm or superficially porous particles under 3 µm (101). Recent studies have applied this technique to successfully characterize isomeric urolithin glucuronides and lignans derived from softwood species (102, 103).

## 4 Bioavailability of polyphenols

Bioavailability refers to the proportion of polyphenol-derived nutrients that are consumed, absorbed, and metabolized (104, 105). Several factors influence the bioavailability of polyphenols, including gut microbiota, nutritional matrix, molecule size, sex, previous dietary habits, transmembrane transport capacity, and chemical structure (106, 107). Additionally, polyphenols interact with gut microbial strains, which can alter their molecular states and affect their subsequent bioactivity (106).

Polyphenols are also subject to various denaturing conditions (103), such as heat, light, oxygen, pH variations, and enzymatic degradation, which reduce their bioavailability and limit their efficacy as bioactive compounds (108). Their bioavailability varies depending on their chemical forms, such as esters, glycosides, or polymers (104). Gao et al. (86) reported that after digestion, the bioavailability of phenolic compounds in *Cannabis sativa* L. seeds was 142.39%, whereas that of flavonoid compounds was 29.47%.

Sánchez-Velázquez et al. (105) revealed that phenolic compounds from wild blackberries might exhibit greater bioactivity and bioavailability in the human body than those from commercial varieties. Similarly, Frazzini et al. (109) examined the effect of *in vitro* gastrointestinal digestion on the bioavailability and stability of polyphenols in commercial and wild Mexican blackberries. Other studies have demonstrated that polyphenols are more stable in organic solvents and water than in cell culture media, where they degrade more rapidly (110). This suggests that polyphenols are prone to degradation in biological systems, potentially reducing their bioavailability and biological efficacy (110–112).

Generally, most dietary polyphenols undergo hydrolysis by colonic bacteria and are then methylated and conjugated into glucuronide and sulfate metabolites by the hepatic and other tissues (106). An increase in plasma antioxidant capacity following the intake of polyphenol-rich foods, such as apples, tea, blackcurrants, and red wine, indicates that polyphenols can cross the intestinal barrier and exert systemic effects (113). Bioavailability has also been directly assessed by measuring polyphenol concentrations in plasma and urine after ingestion of purified compounds or polyphenol-rich foods (114). However, despite their health benefits, the low absorption rate of polyphenols (approximately 5–10% via the small intestine) and their rapid metabolism and excretion significantly limit their ability to reach target tissues (114). Kou et al. (115) reported that purified blueberry polyphenol extract exhibited higher antioxidant activity in different *in vitro* assays, whereas the crude blueberry extract demonstrated greater antioxidant effectiveness in *in vivo* models.

To improve the bioavailability of polyphenols, an investigation (116) was conducted to evaluate their stability in sports nutritional products incorporating both plant polyphenols and milk proteins. A study by van de Langerijt (116) examined the potential of integrating these components into sports supplements to preserve polyphenol content and enhance bioavailability during digestion (116). It showed that anthocyanins remained stable during *in vitro* digestion, with enhanced bioavailability observed in milk-blackberry mixtures, particularly those made with full-cream milk (116). Another study evaluated the bioavailability of total polyphenols from coffee silver skin extract using simulated gastrointestinal digestion and colonic fermentation (117). The findings suggested that fermentation enhanced antioxidant activity and enabled delivery to target sites, supporting potential health benefits (117).

Heat treatment has also been shown to improve polyphenol stability and bioactivity. Franková et al. (118) reported that heat processing of sweet potatoes enhanced both their antioxidant capacity and phenolic content. These findings indicate that thermal processing enhances the bioavailability of polyphenols in sweet potatoes and may guide advancements in food processing technologies. Furthermore, nanoencapsulation techniques, such as incorporating polyphenols into nanoparticles (NPs) or liposomes, can further improve their bioavailability and biological activity (108).

Despite significant progress in understanding polyphenol bioavailability, several gaps and future research priorities remain. One major limitation is the incomplete knowledge of the metabolic pathways and transformations that polyphenols undergo after ingestion, particularly due to interactions with the gut microbiome and the formation of diverse metabolites whose biological activities are not well characterized (119). Additionally, the influence of food processing, individual genetic variability, and the complex interactions between polyphenols and other dietary or environmental components on their absorption and bioactivity requires further exploration (120).

There is also a critical need for well-designed long-term safety studies addressing the potential side effects of chronic polyphenol supplementation, as current data are mainly limited to short-term animal experiments or isolated compounds, and results from these do not always translate directly to humans (121). While some polyphenol-rich extracts, such as grape seed extract, have shown high tolerability in animal and short-term human studies, the safety of long-term, high-dose intake across a broad population spectrum remains to be confirmed (122, 123). Special attention should be given to possible interactions with medications, effects on nutrient absorption (such as iron), and risks to sensitive populations (121, 124, 125).

Clinical research on polyphenol bioavailability is advancing, but large-scale intervention trials remain scarce. More chronic, placebo-controlled human studies are required to evaluate not only the bioavailability and efficacy of various polyphenol formulations and delivery systems but also to establish standardized dosages, monitor potential side effects, and assess inter-individual differences in responses due to genetics and gut microbiota composition (126, 127). The development and validation of robust biomarkers for polyphenol intake and metabolism are also needed to improve accuracy in such studies (127).

Future studies should focus on enhancing the understanding of the metabolic pathways of polyphenols and the bioactivity of their metabolites. It is necessary to expand extensive chronic clinical trials to evaluate the long-term safety, efficacy, and optimal dosing of polyphenols, examine gene-diet and microbiota-polyphenol interactions to elucidate inter-individual variability, develop innovative delivery systems to improve bioavailability and facilitate clinical translation, and clarify potential drug interactions and safety in vulnerable populations.

### 4.1 Bioavailability of polyphenols encapsulated in liposomes or NPs and their functional impact

Encapsulation is a delivery mechanism that incorporates bioactive compounds, such as drugs or food ingredients, into carrier systems (108). This approach protects the active substances from degradation during processing and storage while increasing their bioactivity by facilitating targeted delivery to specific organs or tissues (108). Despite their potential, polyphenols remain underutilized in functional foods and dietary supplements due to several physicochemical properties, including low epithelial permeability, poor solubility in gastrointestinal fluids, structural modifications during digestion, and limited oral bio-accessibility (128, 129).

To overcome these challenges, various technologies have been developed to improve the bioavailability of polyphenols, with nanoencapsulation and liposomal encapsulation considered the most effective strategies. Effective delivery of bioactive compounds to target sites requires a reduction in particle size (130). Nanoencapsulation, typically within a diameter range of 10 to 1000 nm, enhances bioavailability, protects against degradation, and enables precise delivery of polyphenols to targeted sites (130). Liposomal encapsulation is an advanced method designed to stabilize sensitive bioactive compounds (131). It supports the encapsulation of both hydrophobic and hydrophilic molecules, thereby optimizing nutrient absorption and biological efficacy. Lipid- and water-based vesicles enhance solubility and membrane permeability, facilitating accurate delivery to the target tissues (131). Furthermore, lipophilic complexes facilitate intestinal absorption while shielding polyphenols from adverse interactions or breakdown during the digestive process (131).

These encapsulation technologies have shown promising potential in improving the bioavailability and biological activity of polyphenols. Ali et al. (132) demonstrated that grape seed extract encapsulated in liposomes exhibited anti-aging, skin-brightening, and moisturizing effects in human skin cells. These findings advocate the development of more soluble and aesthetically desirable formulations for a broad range of skincare products (133). Altan et al. (134) conducted a study to promote bone wound healing in a rat model using a liposomal formulation of gallic acid. The study included four groups of rats. The group treated with gallic acid liposomes showed the greatest improvement and the lowest infection rate, whereas the negative control group exhibited the least improvement and the highest infection rate (134). These findings indicate that liposomal encapsulation improves the bioavailability and bioactivity of gallic acid polyphenols (134).

Previous research on polyphenols from various plant sources encapsulated in nanoliposomes has shown that increased bioavailability correlates with enhanced antimicrobial activity (135, 136). For instance, Nateghi et al. (135) assessed the antimicrobial activity of phenolic compounds from *Achillea millefolium* encapsulated in nanoliposomes against *Campylobacter jejuni* infection in mice. The study demonstrated that nanoencapsulated polyphenols significantly enhanced antioxidant levels, hepatic function, and food consumption compared to nonencapsulated treatments (135). Furthermore, the proliferation of *C. jejuni* was markedly reduced in mice receiving nano-encapsulated polyphenols, supporting the potential use of polyphenol-loaded nanoliposomes as phytobiotics against this infection (135).

In a similar study, Hassirian et al. (136) investigated the dietary phytobiotic effects of the phenolic-rich fraction of *Alcea rosea* against *Escherichia coli* infection in mice. The study aimed to assess the antimicrobial and potential health-promoting properties of phenolic-rich nanoliposomes, which showed greater efficacy compared to unencapsulated polyphenols at the same dosage (136). Furthermore, Shamansoori et al. (137) demonstrated that an extract of *Rheum ribes* encapsulated in nanoliposomes acted as a novel phytogenic antibiotic, effectively protecting mice from *E. coli* infection. Encapsulated polyphenols (10 mg TPC/kg) significantly improved health markers in mice compared to non-encapsulated forms (137).

Similarly, Mehdizadeh et al. (138) reported comparable results using *Artemisia aucheri* phenolic compounds encapsulated in nanoliposomes to treat *C. jejuni* infection in mice. *In vivo* studies also demonstrated the protective effects of liposome-encapsulated ferulic acid against oxidative liver damage (139). Encapsulated ferulic acid exhibited antioxidant properties by reducing CCl_4_-induced cytotoxicity *in vitro* and significantly alleviated hepatotoxicity, ROS production, and tissue damage in rat liver following intravenous administration (139).

Another animal study reported that liposome-encapsulated p-coumaric acid (CA) inhibited osteoclast formation and bone resorption in a rat model of rheumatoid arthritis, suggesting its potential to prevent bone degradation and calcium loss (140). A study on broiler breeder roosters investigated the effect of ellagic acid-loaded liposomes on post-thaw sperm quality (141). Results indicated that 1 mM ellagic acid liposomes significantly improved sperm antioxidant levels and overall quality after thawing. Furthermore, research on green tea polyphenols in photodynamic cancer therapy demonstrated that NPs of these polyphenols induced higher apoptotic rates and more potent inhibition of cancer cell proliferation than non-NP forms (142). This underscores the role of nanomedicine in enhancing the ‘anti-tumor’ bioactivity and bioavailability of green tea polyphenols (142). Additionally, the anti-cancer effects of silk fibroin NPs encapsulating rosmarinic acid (RA), a polyphenol with antimicrobial, antioxidant, and other bioactivities, were investigated in HeLa and MCF-7 cell lines. The study concluded that NPs improve the solubility and bioavailability of RA, thereby augmenting its anti-cancer efficacy (143).

Zhu et al. (144) enhanced the anti-cancer efficacy of curcumin NPs. Curcumin, a potent phenolic compound, exhibits various physiological effects, including anti-inflammatory, antioxidant, and ‘anti-tumor’ properties. However, its application is limited by volatility and poor buccal bioavailability. Moreover, curcumin was encapsulated into pea protein using a pH-driven NP method. This method yielded curcumin-loaded pea protein NPs with significantly higher loading efficiency and improved water solubility (144). In a separate study, potent antioxidative and ‘anti-tumor’ NPs were synthesized from tea polyphenols using an amino acid-induced ultrafast method (145). Epigallocatechin gallate (EGCG), a primary antioxidant in green tea, was used to prepare a therapeutic nano-agent via a rapid process involving five amino acids: lysine, arginine, leucine, glycine, and glutamic acid. The study found that lysine and arginine were depleted within 50 seconds of induction. The resulting NPs displayed tenfold greater antioxidant activity than conventional NPs and demonstrated therapeutic efficacy against cancer in both *in vitro* and *in vivo* models (145). Another study utilized *Punica granatum* (pomegranate) extract for the green synthesis of silver NPs (146). Silver NPs synthesized from a polyphenol-rich fraction exhibited antimicrobial activity against *Staphylococcus aureus*, *Bacillus subtilis*, and *Sarcina lutea* (146).

These findings suggest that encapsulation enhances the bioactivity, solubility, and permeability of polyphenols by increasing their bioavailability. However, further studies are required to demonstrate that encapsulation improves the biological efficacy of polyphenols conclusively.

## 5 Health benefits of polyphenols

The inclusion of polyphenol-rich foods and beverages, including tea, herbs, fruits, and wine, in the diet is an effective approach to harness their health-promoting properties (147, 148). Polyphenols exhibit a wide range of biological activities, including anti-inflammatory, anti-diabetic, antimicrobial, antioxidant, anti-aging, anti-cancer, and cytotoxic properties (149, 150). These properties contribute to the prevention of chronic diseases and support therapeutic strategies. Furthermore, polyphenols have demonstrated positive effects on cardiovascular health and cognitive function, potentially through the prevention of neurodegenerative disorders (151).

**Figure 2:**
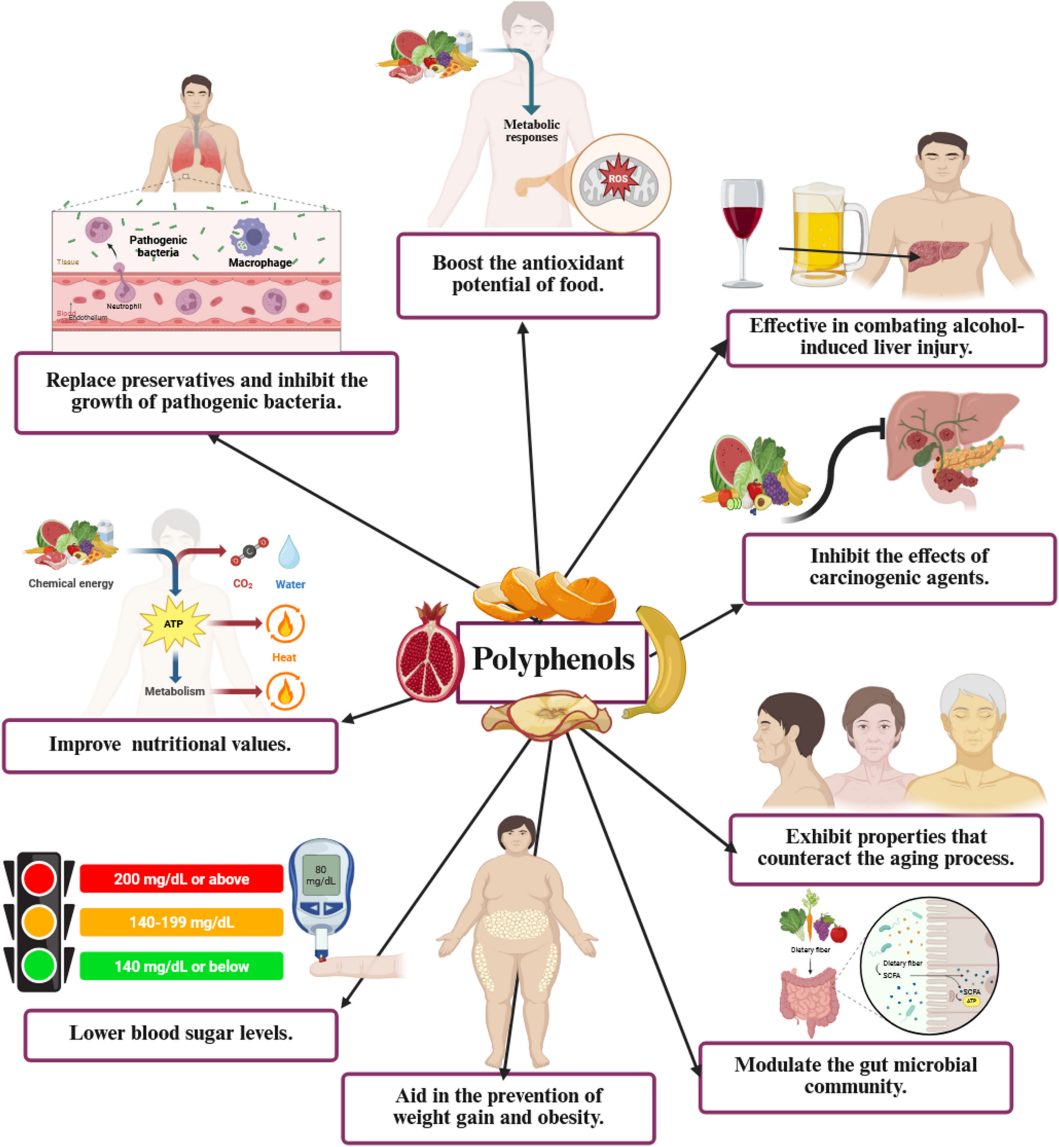
Comprehensive health advantages of polyphenols: A visual depiction of their functions in enhancing antioxidant capacity, mitigating alcohol-related liver damage, suppressing carcinogenic effects, countering aging, regulating gut microbiota, facilitating weight management and obesity prevention, reducing blood glucose levels, augmenting nutritional value, and substituting preservatives by inhibiting pathogenic bacterial proliferation. ROS, reactive oxygen species.

**Figure 2** highlights the diverse health advantages of polyphenols: a visual depiction of their functions in enhancing antioxidant capacity, mitigating alcohol-related hepatic damage, obstructing carcinogenic effects, retarding the aging process, regulating gut microbiota, facilitating weight management and obesity prevention, reducing blood glucose levels, augmenting nutritional value, and substituting preservatives by inhibiting pathogenic bacterial proliferation.

### 5.1 Antioxidant activity

One of the most extensively studied properties is their antioxidant activity. A key function of polyphenols is their ability to reduce or prevent ROS, which are harmful to human health (152, 153). By neutralizing ROS, polyphenols exert protective effects against oxidative stress and skin degradation (154, 155). Polyphenols interact with ROS primarily through three mechanisms governed by their molecular structure: single electron transfer, hydrogen atom transfer, and transition metal chelation (156, 157). In the hydrogen atom transfer mechanism, polyphenols donate a hydrogen atom from their phenolic hydroxyl group, producing free radicals that neutralize ROS (156). The efficiency of this reaction is associated with the bond dissociation enthalpy of the O–H bond; lower bond dissociation enthalpy corresponds to higher reactivity. For instance, in the reaction R + ArOH → ArO + RH, a lower bond dissociation enthalpy facilitates hydrogen donation (158, 159).

In the single electron transfer mechanism, antioxidant capacity is related to ionization potential; molecules with low ionization potential values act as efficient electron donors in the reaction R + ArOH → R^–^ + ArOH^+^ → RH + ArO (158). In the transition metal chelation mechanism, polyphenol anions chelate heavy metals through the deprotonation of hydroxyl groups, forming metal complexes and releasing a proton (ArOH → ArO^–^ + H+) (160). These three pathways collectively evaluate the antioxidant potential of polyphenols in protecting human health against oxidative damage (157).

Different polyphenols exert distinct effects on antioxidant activity (161, 162). For instance, quercetin has demonstrated potent antioxidant properties (163). The antioxidant efficacy of polyphenols has been extensively investigated in both *in vivo* and *in vitro* studies (164, 165), confirming their preventive role against various diseases (166, 167). The pharmacological potential of *Rhododendron tomentosum* has been linked to its polyphenolic composition, including chlorogenic acid, caffeic acid, rutin, and quercetin, as identified by high-performance thin-layer chromatography (168).

The antioxidant activities of these compounds were confirmed using a 2,2-diphenyl-1-picrylhydrazyl (DPPH) radical-scavenging assay. Similarly, the DPPH assay was used to evaluate the antioxidant activity of Zhourat plants (169). Following the quantification of total phenolic acids, different solvent extractions were assessed for free radical scavenging capacity. Water/ethanol extracts often exhibit superior antioxidant activity compared to pure water or ethanol-only extracts, with two exceptions (169). Bashmil et al. (170) showed that unripe bananas possessed higher free radical scavenging ability than ripe ones, highlighting the influence of polyphenol type, structure, and phenolic ring count on antioxidant efficacy. Janarny et al. (171) examined the antioxidant capacity of edible flowers from the family Fabaceae (171). In *Chamanerion angustifolium* L. (fireweed) leaves, antioxidant activity varied with fermentation conditions. Notably, activity decreased after 24 h of fermentation under both aerobic and anaerobic conditions; however, it increased after 48 h compared to unfermented leaves (171).

Bobkova et al. (172) evaluated the antioxidant potential of coffee using free radical-scavenging methods, revealing that antioxidant capacity varied with geographical origin due to differences in polyphenol content (172). Alsaud et al. (173) reported that *Leptospermum scoparium* (Manuka) leaves exhibited significant ferric-reducing antioxidant power (FRAP assay) and free radical scavenging activity (DPPH assay) (173). The ethanolic extract outperformed most deep eutectic solvent extracts, though some deep eutectic solvent extracts exhibited higher ferric-reducing antioxidant power values. Overall, polyphenols exhibit antioxidant properties through various pathways, including free radical scavenging and the augmentation of endogenous antioxidant enzyme activity (174, 175).

**Figure 3:**
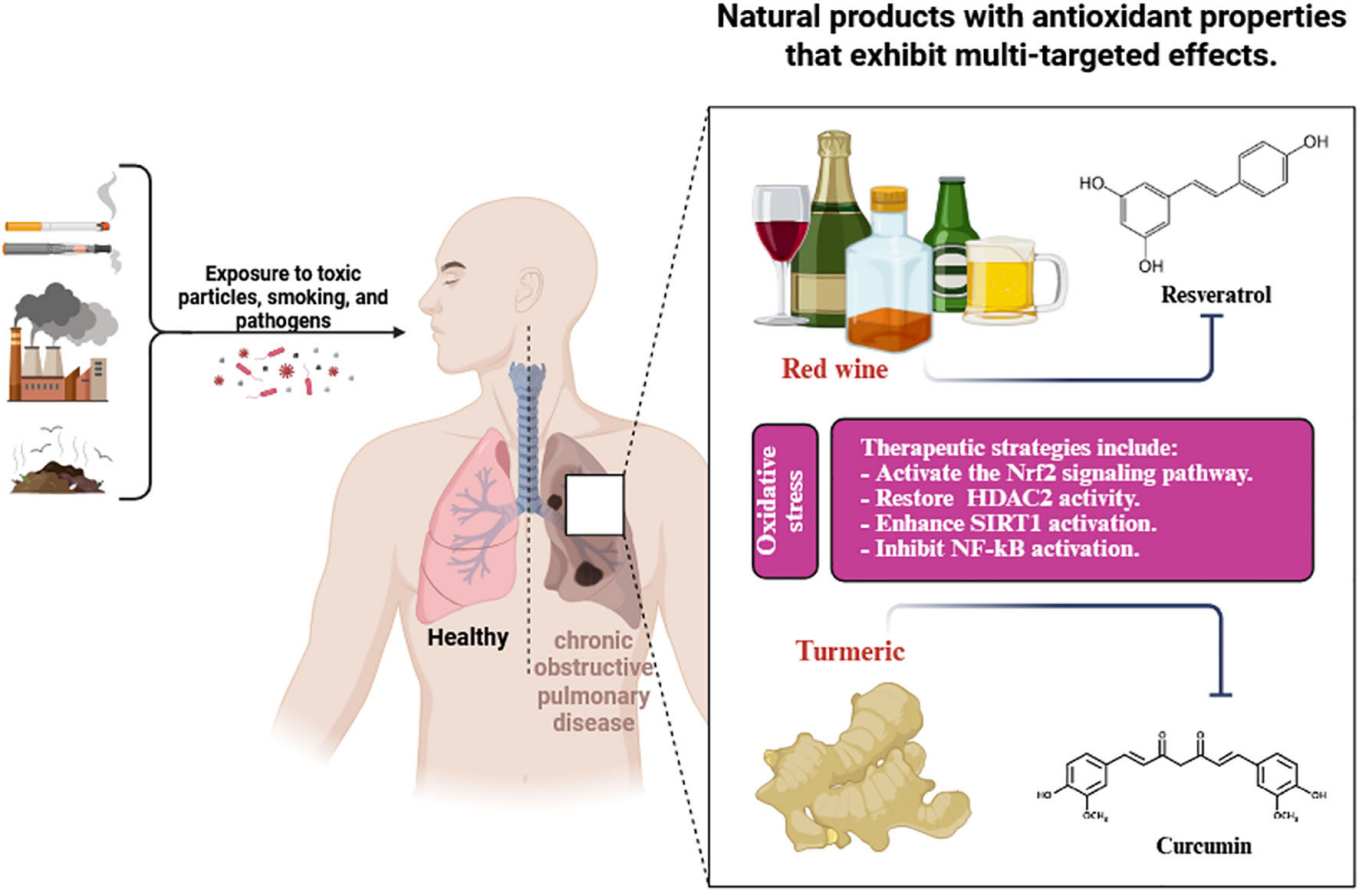
Antioxidant properties of natural compounds (resveratrol from red wine and curcumin from turmeric) as therapeutic approaches to combat oxidative stress-induced chronic obstructive pulmonary disease caused by exposure to harmful particles, smoking, and infections.

**Table 1** illustrates the sources, classifications, antioxidant efficacy, and modes of action of polyphenols. **Figure 3** illustrates the antioxidant properties of natural compounds, specifically resveratrol from red wine and curcumin from turmeric, as therapeutic interventions for oxidative stress-induced chronic obstructive pulmonary disease caused by exposure to harmful particles, smoking, and infections.

**Table 1:** Diverse sources and types of polyphenols, their antioxidant properties, and modes of action.

Polyphenol sources	Polyphenol/s	Antioxidant activities	Mode of action	References
Green tea	Catechins (Flavonoids)	Exhibits vigorous antioxidant activity, scavenging free radicals and reducing oxidative stress	Direct scavenging of reactive oxygen species and upregulation of antioxidant enzymes	(176, 177)
Red wine	Resveratrol (Stilbene)	Demonstrates antioxidant properties, protecting cells from oxidative damage	Scavenges reactive oxygen species and modulates antioxidant enzyme expression	(178, 179)
Dark chocolate	Flavonoids	Possesses antioxidant effects, reducing oxidative stress and improving vascular health	Neutralizes free radicals and enhances endothelial function	(180, 181)
Blueberry	Anthocyanins (Flavonoids)	Exhibits antioxidant activity, protecting against DNA damage	Scavenges free radicals and chelates metal ions	(182, 183)
Turmeric	Curcumin (Diferuloylmethane)	Shows potent antioxidant properties, protecting cells from oxidative damage	Neutralizes free radicals and enhances the activity of antioxidant enzymes	(184–186)
Oat	Avenanthramides	Exhibits antioxidant activity, protecting against oxidative stress	Scavenges free radicals and inhibits oxidative enzymes	(187, 188)
Grape	Resveratrol (Stilbene)	Demonstrates antioxidant properties, protecting cells from oxidative stress	Scavenges reactive oxygen species and modulates antioxidant enzyme expression	(189, 190)
Apple	Quercetin (Flavonoid)	Exhibits antioxidant activity, reducing oxidative stress	Scavenges free radicals and inhibits lipid peroxidation	(191, 192)
Pomegranate	Punicalagins (Ellagitannins)	Shows strong antioxidant properties, protecting cells from oxidative damage	Scavenges free radicals and inhibits oxidative enzymes	(193, 194)
Cranberry	Proanthocyanidins (Flavonoids)	Demonstrates antioxidant activity, reducing oxidative stress	Scavenges free radicals and chelates metal ions	(195, 196)
Strawberry	Anthocyanins (Flavonoids)	Exhibits antioxidant properties, protecting against oxidative damage	Scavenges free radicals and inhibits lipid peroxidation	(197, 198)
Roseberry	Ellagic acid (Phenolic Acid)	Shows antioxidant activity, reducing oxidative stress	Scavenges free radicals and modulates antioxidant enzymes	(199, 200)
Blackberry	Anthocyanins (Flavonoids)	Possesses antioxidant properties, protecting cells from oxidative damage	Scavenges free radicals and chelates metal ions	(201, 202)
Cherry	Anthocyanins (Flavonoids)	Demonstrates antioxidant activity, reducing oxidative stress	Scavenges free radicals and inhibits oxidative enzymes	(203, 204)
Spinach	Flavonoids	Exhibits antioxidant properties, protecting against oxidative damage	Scavenges free radicals and enhances antioxidant defenses	(205, 206)
Kale	Flavonoids	Shows antioxidant activity, reducing oxidative stress	Scavenges free radicals and modulates antioxidant enzymes	(207, 208)
Broccoli	Flavonoids	Possesses antioxidant properties, protecting cells from oxidative damage	Scavenges free radicals and inhibits oxidative enzymes	(209, 210)
Onion	Quercetin (Flavonoid)	Demonstrates antioxidant activity, reducing oxidative stress	Scavenges free radicals and inhibits lipid peroxidation	(211, 212)
Tomato	Lycopene (Carotenoid)	Exhibits antioxidant properties, protecting against oxidative damage	Scavenges free radicals and enhances antioxidant defenses	(213, 214)
Carrot	Beta-Carotene (Carotenoid)	Shows antioxidant activity, reducing oxidative stress	Scavenges free radicals and modulates antioxidant enzymes	(215, 216)
Orange	Hesperidin (Flavanone)	Possesses antioxidant properties, protecting cells from oxidative damage	Neutralizes free radicals and suppresses oxidative enzymes	(217, 218)
Lemon	Eriocitrin (Flavanone)	Demonstrates antioxidant activity, reducing oxidative stress	Scavenges free radicals and enhances antioxidant defenses	(219, 220)
Soybean	Isoflavones	Exhibits antioxidant properties, protecting against oxidative damage	Scavenges free radicals and modulates antioxidant enzymes	(221, 222)
Red Onion	Quercetin (Flavonoid)	Shows antioxidant activity, reducing oxidative stress	Scavenges free radicals and inhibits lipid peroxidation	(223, 224)

### 5.2 Anti-inflammatory activity

The hydroxyl groups and unique aromatic ring structures of polyphenols allow them to exert regulatory effects on various inflammatory pathways (225, 226). Polyphenols can suppress the expression and activity of key pro-inflammatory mediators, such as nuclear factor-κB (NF-κB), a transcription factor vital to the regulation of the inflammatory response, as shown in **Table 2** (269, 270).

**Table 2:** Different polyphenol sources, types, anti-inflammatory effects, and modes of action.

Polyphenol sources	Polyphenol/s	Anti-inflammatory activities	Mode of action	References
Turmeric	Curcumin	Reduces symptoms of inflammatory bowel disease, stomach ulcers, and Crohn’s disease	Inhibits nuclear transcription factor kappa B (NF-κB) activation, and reduces pro-inflammatory cytokine production	(227, 228)
Green tea	Catechins (Flavonoids)	Exhibits anti-inflammatory properties, aiding in reducing inflammation	Reduces oxidative stress and modulates inflammatory pathways	(229–231)
Red wine	Resveratrol (Stilbene)	May offer health benefits due to its anti-inflammatory properties	Inhibits pro-inflammatory mediators and modulates inflammatory signaling pathways	(179, 232)
Oat	Avenanthramides	Significantly reduces the inflammatory response	Inhibits NF-κB activation, reducing pro-inflammatory cytokine production	(233, 234)
Elderberry	Anthocyanins (Flavonoids)	Reduces inflammation and supports immune health	Modulates inflammatory pathways and reduces pro-inflammatory cytokine production	(235, 236)
Dark chocolate	Flavonoids	Rich in flavonoids and polyphenols, aiding in reducing inflammatory stress	Scavenges reactive oxygen species and modulates inflammatory pathways	(237, 238)
Olive oil	Oleocanthal (Phenolic compound)	Contains oleocanthal and monounsaturated fats, beneficial for reducing inflammation	Inhibits cyclooxygenase enzymes and reduces prostaglandin synthesis	(239, 240)
Grape	Resveratrol (Stilbene)	Exhibits anti-inflammatory properties	Inhibits pro-inflammatory mediators and modulates inflammatory signaling pathways	(241, 242)
Berry	Anthocyanins (Flavonoids)	Reduces inflammation and oxidative stress	Scavenges reactive oxygen species and inhibits pro-inflammatory cytokine production	(243, 244)
Tomato	Lycopene (Carotenoid)	Rich in lycopene, which intensifies with cooking, perfect for sauces and soups	Scavenges reactive oxygen species and modulates inflammatory pathways	(213, 245)
Garlic	Allicin (Organosulfur compound)	Exhibits anti-inflammatory properties	Inhibits pro-inflammatory enzymes and cytokine production	(246, 247)
Onion	Quercetin (Flavonoid)	Reduces inflammation and supports immune health	Inhibits pro-inflammatory enzymes and cytokine production	(248, 249)
Broccoli	Sulforaphane (Isothiocyanate)	Exhibits anti-inflammatory properties	Activates Nrf2 pathway, enhancing antioxidant response and reducing inflammation	(250, 251)
Spinach	Flavonoids	Reduces inflammation and oxidative stress	Scavenges reactive oxygen species and modulates inflammatory pathways	(205, 206)
Kale	Flavonoids	Exhibits anti-inflammatory properties	Scavenges reactive oxygen species and modulates inflammatory pathways	(252, 253)
Sweet potato	Beta-Carotene (Carotenoid)	Reduces inflammation and supports immune health	Scavenge reactive oxygen species and modulates inflammatory pathways	(254, 255)
Purple corn	Anthocyanins (Flavonoids)	Exhibits anti-inflammatory properties	Scavenges reactive oxygen species and inhibits pro-inflammatory cytokine production	(256, 257)
Microgreen	Flavonoids	Reduces inflammation and oxidative stress	Scavenges reactive oxygen species and modulates inflammatory pathways	(258, 259)
Pepper	Capsaicin (Capsaicinoid)	Exhibits anti-inflammatory properties	Inhibits pro-inflammatory neuropeptides and cytokine production	(260, 261)
Mushroom	Ergothioneine (Antioxidant)	Reduces inflammation and supports immune health	Scavenges reactive oxygen species and modulates inflammatory pathways	(262, 263)
Chayote	Flavonoids	Exhibits anti-inflammatory properties	Scavenges reactive oxygen species and inhibits pro-inflammatory cytokine production	(263, 264)
Avocado	Polyphenols	Reduces inflammation and supports heart health	Scavenges reactive oxygen species and modulates inflammatory pathways	(265, 266)
Carrot	Beta-Carotene (Carotenoid)	Exhibits anti-inflammatory properties	Scavenges reactive oxygen species and modulates inflammatory pathways	(267, 268)

By inhibiting the activation of NF-κB, polyphenols reduce the expression of pro-inflammatory genes and the synthesis of inflammatory cytokines and enzymes (271, 272). Additionally, polyphenols may modulate the biosynthesis of pro-inflammatory lipid mediators by affecting the enzymatic activities involved in inflammation, thereby contributing to their anti-inflammatory action (270, 273).

Polyphenols also regulate immune cell function by modulating the activity of dendritic cells, lymphocytes, and macrophages (274, 275). Moreover, they influence immune recruitment and migration by altering the synthesis of chemokines and adhesion molecules (271, 276). Synergistic interactions among various polyphenolic compounds may further enhance their anti-inflammatory effects (277). A comparative analysis of polyphenols extracted from celery, coriander, and parsley revealed that celery had the highest total polyphenol content, followed by coriander and parsley (278). However, parsley polyphenols demonstrated the most potent nitric oxide scavenging activity, which is essential in inflammation due to the overproduction of nitric oxide. When tested for their ability to prevent the protein denaturation effect, parsley extract again showed superior activity (278). Similarly, in membrane stabilization assays, used to assess the protection of erythrocyte membranes under inflammatory stress, parsley extract demonstrated superior activity (278). Polyphenolic compounds in berries have also been extensively studied for their anti-inflammatory properties (279, 280).

Kim et al. (281) reported that polyphenols from black raspberry roots significantly inhibited the production of nitric oxide and prostaglandin E2 in lipopolysaccharide (LPS)-activated RAW264.7 macrophages in a dose-dependent manner. These root polyphenols were more effective than those from unripe fruits in reducing the levels of pro-inflammatory cytokines and downregulating the mRNA expression of cyclooxygenase-2 (COX-2) and inducible nitric oxide synthase (281).

Furthermore, these polyphenols exhibited strong antimicrobial activity against methicillin-resistant *Bacillus anthracis, S. aureus* (MRSA), and carbapenem-resistant *Acinetobacter baumannii*. Peng et al. (282) demonstrated that polyphenol-rich extracts from rice wine significantly downregulated inducible nitric oxide synthase expression and reduced nitric oxide production. The extracts also suppressed the expression of pro-inflammatory cytokines, including tumor necrosis factor-alpha (TNF-α), interleukin-6 (IL-6), and interleukin-1 beta (IL-1β) (282). These effects were associated with the inhibition of NF-κB nuclear translocation and reduced phosphorylation of κB and mitogen-activated protein kinases, including p38, extracellular signal-regulated kinases 1 and 2 (Erk 1/2), and c-Jun N-terminal kinase (282). Zhang et al. (283) found that polyphenols inhibited nitric oxide production and reduced the expression of IL-1β, IL-6, TNF-α, and nitric oxide synthase in LPS-activated macrophages. These compounds also suppressed NF-κB activation and mitogen-activated protein kinases phosphorylation (extracellular signal-regulated kinases 1 and 2 (Erk 1/2), and c-Jun N-terminal kinase).

A study by de Araújo (284) involved the determination of minimum inhibitory concentration (MIC) and agar well-diffusion assays against methicillin-resistant strains of *S. aureus, Salmonella enteritidis, E. coli, Enterococcus faecalis*, and *Staphylococcus epidermidis* (284). Polyphenols from *Psidium guajava* exhibited the largest zones of inhibition in the agar diffusion test. Notably, the polyphenol extracts were more effective against Gram-positive bacteria and ineffective against Gram-negative strains (284).

Anti-inflammatory activity was further evaluated using the carrageenan-induced peritonitis model in mice. Administration of plant extracts significantly reduced the inflammatory response induced by carrageenan (284). However, in acetic acid-induced writhing and analgesic tests, the extracts did not exhibit significant pain-relieving effects, suggesting selective anti-inflammatory rather than analgesic activity (284). These findings support the potential of these plant-derived polyphenols in managing inflammatory conditions (283, 284).

Fermentation plays a significant role in modifying the bioactivity of polyphenol-rich plant materials (284, 285). Recent research by Sim et al. (286) showed that complex fermentation of *Pyrus montana* and *Maclura tricuspidata* using lactic acid bacteria, yeast, and *Aspergillus shirousamii* enhanced their phenolic content and anti-inflammatory activity. Fermented extracts exhibited increased DPPH and ABTS radical-scavenging capacities and significantly reduced nitric oxide production from day six of fermentation. Western blot analysis revealed suppression of TNF-α, COX-2, and nitric oxide synthase protein expression, indicating effective inhibition of inflammation-related signaling pathways. Overall, polyphenols from various plants, algae, and natural sources possess notable anti-inflammatory potential and contribute to the prevention and management of chronic inflammatory diseases (287, 288). They also offer protective effects against metabolic disorders through their ability to regulate inflammatory signaling pathways (289).

**Table 2** illustrates various sources and types of polyphenols, along with their anti-inflammatory properties and modes of action.

### 5.3 Antimicrobial activity

Antimicrobial activity refers to the ability of a substance to inhibit or reduce the growth of microorganisms, including bacteria, viruses, parasites, and fungi (290, 291). Antimicrobial agents are widely used in medicine, agriculture, and the food industry to combat microbial infections (292, 293). As shown in **Table 3**, the antibacterial properties of plant extracts are attributed mainly to their phenolic compounds (337, 338).

**Table 3:** Numerous polyphenol sources, categories, antimicrobial properties, and mechanisms of action.

Polyphenol sources	Polyphenol/s	Antimicrobial activities	Mechanism of actions	References
Green tea	Catechins (Flavonoids)	Exhibits antibacterial activity against various pathogens, including *Escherichia coli* and *Staphylococcus aureus*	Disruption of bacterial cell membranes and inhibition of enzyme activity	(294–296)
Black tea	Theaflavins (Flavonoids)	Possesses antimicrobial properties that may help prevent diarrhea and influence gut microbiota	Inhibition of bacterial enzymes and interference with microbial metabolism	(296, 297)
Turmeric	Curcumin (Diferuloylmethane)	Demonstrates broad-spectrum antimicrobial activity against bacteria, fungi, and viruses	Disruption of microbial cell membranes and inhibition of nucleic acid synthesis	(298, 299)
Cranberry	Proanthocyanidins (Flavonoids)	May reduce the incidence of urinary tract infections by inhibiting bacterial adhesion	Prevention of bacterial adhesion to urinary tract walls	(300, 301)
Grape	Resveratrol (Stilbene)	Exhibits antifungal activity against pathogens like *Botrytis cinerea*	Induction of oxidative stress in fungal cells and inhibition of fungal enzymes	(302, 303)
Pine bark	Proanthocyanidins (Flavonoids)	Shows antimicrobial activity against various bacteria and fungi	Disruption of microbial cell walls and inhibition of microbial enzymes	(304, 305)
Pomegranate	Ellagitannins (Tannins)	Exhibits antibacterial activity against *Staphylococcus aureus* and *Escherichia coli*	Disruption of bacterial cell membranes and inhibition of bacterial enzymes	(306, 307)
Olive oil	Hydroxytyrosol (Phenolic compound)	Demonstrates antimicrobial activity against various bacterial strains	Disruption of bacterial cell membranes and inhibition of bacterial growth	(308, 309)
Red wine	Resveratrol (Stilbene)	Exhibits antimicrobial activity against various pathogens	Disruption of microbial cell membranes and inhibition of nucleic acid synthesis	(310, 311)
Blueberry	Anthocyanins (Flavonoids)	Shows antimicrobial activity against various bacterial strains	Disruption of bacterial cell membranes and inhibition of bacterial enzymes	(182, 312)
Cocoa	Flavanols (Flavonoids)	Exhibits antibacterial activity against *Streptococcus mutans*	Inhibition of bacterial glucosyltransferases and prevention of biofilm formation	(313, 314)
Garlic	Allicin (Organosulfur compound)	Demonstrates broad-spectrum antimicrobial activity against bacteria and fungi	Inhibition of microbial thiol-containing enzymes and disruption of cell membranes	(315, 316)
Cinnamon	Cinnamaldehyde (Phenylpropanoid)	Exhibits antimicrobial activity against various bacterial and fungal pathogens	Disruption of microbial cell membranes and inhibition of enzyme activity	(317, 318)
Oregano	Carvacrol (Monoterpenoid phenol)	Shows antimicrobial activity against bacteria such as *Salmonella* and *Escherichia coli*	Disruption of bacterial cell membranes and leakage of cellular contents	(319, 320)
Clove	Eugenol (Phenylpropanoid)	Demonstrates antimicrobial activity against various bacteria and fungi	Disruption of microbial cell membranes and inhibition of enzyme activity	(321, 322)
Thyme	Thymol (Monoterpenoid Phenol)	Exhibits antimicrobial activity against various pathogens	Disruption of microbial cell membranes and inhibition of enzyme activity	(323, 324)
Sage	Rosmarinic Acid (Caffeic acid ester)	Shows antimicrobial activity against bacteria and fungi	Inhibition of microbial enzymes and disruption of cell membranes	(325, 326)
Rosemary	Carnosic Acid (Diterpene)	Demonstrates antimicrobial activity against various bacterial strains	Disrupts bacterial cell membranes and inhibits bacterial growth	(327, 328)
Ginger	Gingerol (Phenolic ketone)	Exhibits antimicrobial activity against various pathogens	Disrupts microbial cell membranes and inhibits the activity of enzymes	(329, 330)
Peppermint	Menthol (Monoterpenoid)	Shows antimicrobial activity against bacteria and fungi	Disrupts microbial cell membranes and inhibits the activity of enzymes	(331, 332)
Licorice	Glycyrrhizin (Saponin)	Demonstrates antimicrobial activity against various bacterial strains	Disrupts bacterial cell membranes and inhibits bacterial growth	(333, 334)
Neem	Azadirachtin (Triterpenoid)	Exhibits antimicrobial activity against various pathogens	Damages bacterial cell membranes and suppresses bacterial growth	(335, 336)

Numerous polyphenols exhibit antimicrobial properties by disrupting cell structures and membranes and interfering with essential enzymatic cellular functions (14, 339). Key determinants of their antimicrobial activity include the presence of carboxyl groups and the arrangement of functional subgroups on the benzene ring (340). Menhem et al. (169) assessed the antimicrobial activity of Zhourat Shamia herbal tea (mixture of herbs and dried flowers) using a disk diffusion assay against foodborne pathogens, including two Gram-positive bacteria (*S. aureus* and *B. cereus*) and two Gram-negative bacteria (*E. coli* and *Pseudomonas aeruginosa*). The phenolic compounds in Zhourat extracts exhibited antimicrobial activity, though efficacy varied depending on the extract and microbial species (169).

A separate study by Gutiérrez-Venegas et al. (341) indicated that rutin, quercetin, and morin had antimicrobial action against *Actinomyces naeslundii* and *Actinomyces viscosus*. While each flavonoid has antimicrobial capabilities against some strains, no antimicrobial impacts have been observed against *Streptococcus oralis* and *Streptococcus sanguinis* (341). The number and type of hydroxyl, carboxyl, and ester groups also play a crucial role by facilitating interactions between polyphenols and microbial cells, thereby inhibiting microbial growth (342, 343). Additionally, polyphenols can interfere with intracellular processes by impairing the activity of enzymes necessary for microbial survival, leading to reduced proliferation (344–346).

De Angelis et al. (347) reported that combinations of polyphenols and micronutrients (A5+) exert antiviral effects against influenza A and SARS-CoV-2. In this study, resveratrol demonstrated antiviral efficacy against respiratory viruses, while polydatin was used as its precursor. Treatment with A5+ and resveratrol significantly reduced SARS-CoV-2 replication. Furthermore, both agents suppressed the expression of essential viral replication proteins and IL-6 in influenza A virus-infected cells. Singh et al. (348) evaluated polyphenols as natural antiviral agents against SARS-CoV-2 using *in silico* analysis, targeting the RNA-dependent RNA polymerase (RdRp) responsible for viral RNA replication. The study found that eight different polyphenols demonstrated favorable binding kinetics, suggesting their potential to inactivate SARS-CoV-2 RdRp (348).

Therefore, polyphenols are considered promising antiviral agents. Musarra-Pizzo et al. (349) conducted antiviral and antimicrobial assays using *Prunus dulcis* L. against *S. aureus* and herpes simplex virus type 1. The antibacterial activity of almonds was inhibited entirely by polyphenols at a concentration of 0.62 mg/mL. Furthermore, antiviral assays revealed that 0.4 mg/mL of almond polyphenols reduced both the expression of viral proteins and the accumulation of viral DNA (349). Park et al. (350) demonstrated that the ethanolic extract of *Aronia melanocarpa*, rich in polyphenols and flavonoids, exhibits antiviral activity. A 0.0625 mg sample of the extract significantly inhibited viral surface proteins in 70% of tested influenza strains, including H1 and H3 subtypes. Pagliarulo et al. (351) evaluated the antimicrobial activity of *Punica granatum* against *S. aureus* and *E. coli*. Pomegranate juice was extracted and then subjected to ethanolic polyphenol extraction of pomegranate using a 50% ethanol/water (v/v) solution. The juice, particularly rich in anthocyanins, was tested in quantities of 1, 2, 4, 8, 10, and 20 mg per disk. The result demonstrated that the extracts inhibited the growth and survival of the tested bacterial strains (351).

Certain extracts exhibited no efficacy against several bacteria, while others exhibited selective antimicrobial effects. Nibir et al. (352) analyzed the total phenolic and flavonoid levels, as well as the antioxidant and antimicrobial properties, of four Chinese tea varieties: broken orange pekoe, black tea, red dust, and green tea. The green tea variety had the highest phenolic and flavonoid content and demonstrated superior antioxidant and antimicrobial activity. The antimicrobial potential of these teas was tested against *Shigella dysenteriae, Shigella boydii, Vibrio cholerae, Salmonella paratyphi, Salmonella typhi, Klebsiella pneumoniae*, and *E. coli* employing agar well-diffusion and MIC assays. These findings confirm that green tea has greater antimicrobial efficacy than the other types (352).

Notably, the antimicrobial activity of polyphenols can be influenced by the extraction procedure and the solvent used (339, 353). Chaudhry et al. (354) examined the effects of extraction methods and solvent systems on yield. Traditional maceration- and ultrasound-assisted extraction techniques were compared using methanol, ethanol, and acetone at 25%, 50%, 75%, and 100% concentrations. Among these, ultrasound-assisted extraction yielded the highest polyphenol content from banana peels (354). Ethanol proved to be the most effective solvent compared to the alternatives. Solvent concentration significantly influenced the yield of polyphenols. Ethanol-based extracts demonstrated superior antioxidant activity, as indicated by the DPPH radical scavenging assay. In contrast, banana peel extracts at various concentrations were tested against *E. coli, P. aeruginosa, S. aureus*, and *Saccharomyces cerevisiae* using the agar disk diffusion method. Measurement of the inhibition zones revealed that ethanol-containing extracts exerted more substantial antimicrobial effects than those obtained with other solvents (354).

In the gut, polyphenols linked to indigestible fibers can contribute to health benefits by releasing bioactive phenolic compounds through microbial fermentation. Thus, incorporating fermentable fiber into the diet may support the growth of beneficial gut microbiota and exert prebiotic effects (355). Although the antimicrobial properties of phenolic compounds are well established, these effects may be modified during gastric digestion (356).

Caponio et al. (357) reported that digestive processes may influence the free radical-scavenging ability of phenolic compounds. Antimicrobial activity was assessed based on effects on the probiotic and pathogenic strains, specifically *Lactiplantibacillus plantarum, Bacillus megaterium, E. coli*, and *Listeria monocytogenes*. These findings indicated that grape pomace-derived polyphenols promoted probiotic growth while inhibiting pathogenic bacteria (357). Similarly, a study on the antimicrobial and digestive behavior of polyphenols from *Hibiscus sabdariffa* showed that these compounds were rapidly released and metabolized in the human digestive tract (358). Polyphenols have demonstrated antimicrobial efficacy against pathogenic bacteria, including L*. monocytogenes* and *S. aureus* (359, 360), making them promising candidates for use as antimicrobial agents (361, 362).

Several *in vivo* studies have confirmed the stability and efficacy of polyphenols following gastrointestinal digestion. For example, dietary supplementation of polyphenol-rich extracts in animal models, such as grape seed extract in broiler chickens and pigs, has been shown to increase the concentration of antioxidant markers like vitamin E in plasma and tissues, suggesting not only the bioavailability but also the effective physiological action of polyphenols after digestion (363). In another study, grape pomace-supplemented feed improved the ratio of polyunsaturated to saturated fatty acids and enhanced the oxidative stability of animal products, indicating that a considerable portion of polyphenols retained their bioactivity after digestive processes (357). Similarly, research has demonstrated that polyphenolic compounds maintain significant antioxidant effects *in vivo*, as evidenced by enhanced plasma antioxidant capacity and reduced markers of oxidative stress in animals supplemented with polyphenols (364).

The findings indicate that, despite specific degradation during digestion, a significant proportion of polyphenols and their metabolites remain sufficiently stable for absorption, hence facilitating their potential health-promoting effects in living organisms post-absorption.

**Figure 4:**
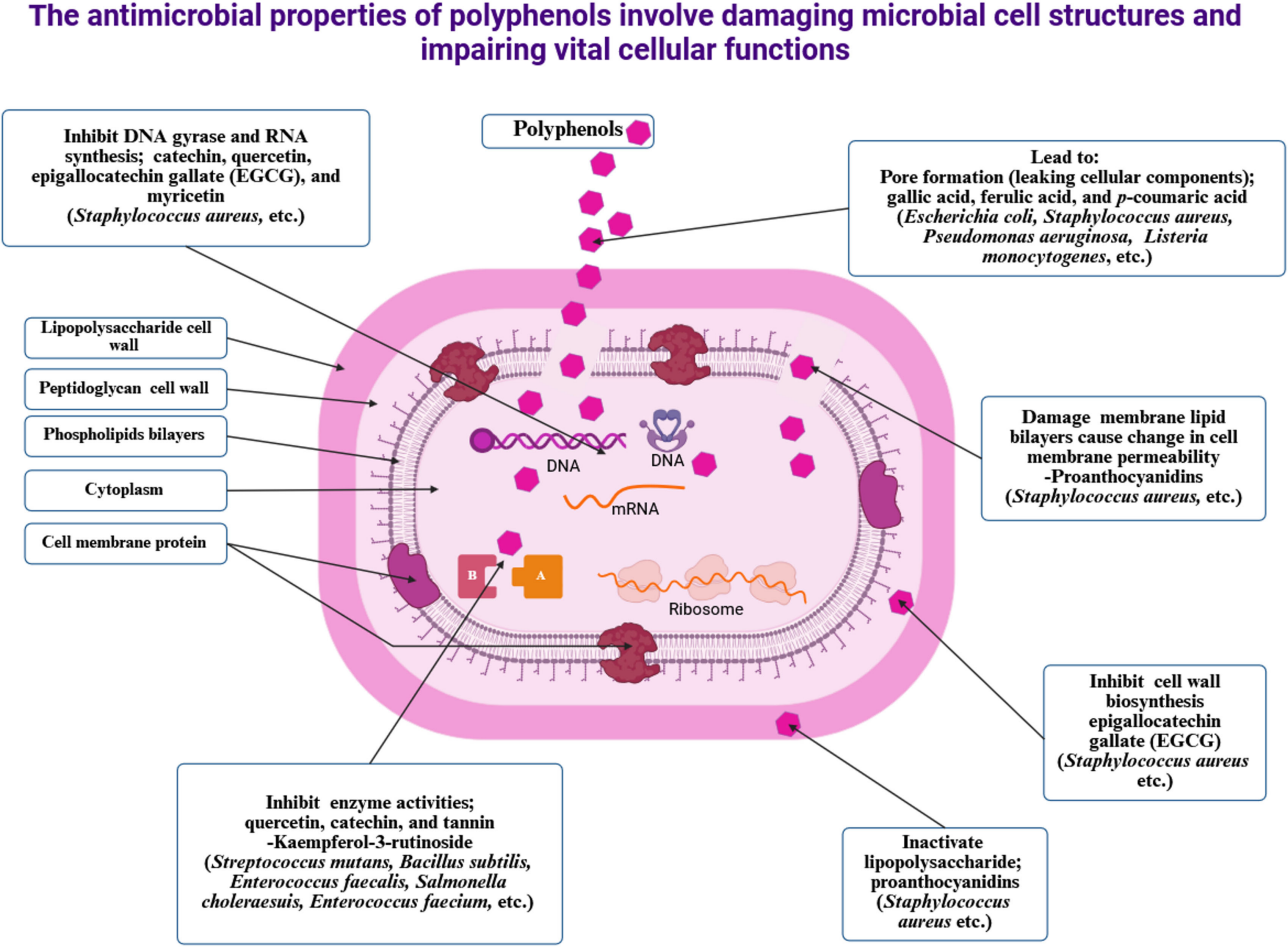
The antimicrobial mechanisms of polyphenols involve the disruption of microbial cell structures, including lipopolysaccharide cell walls, peptidoglycan cell walls, phospholipid bilayers, and cell membrane proteins, as well as the impairment of essential cellular functions such as inhibition of DNA gyrase and RNA synthesis, pore formation causing leakage, damage to membrane lipid bilayers, inhibition of enzyme activity, suppression of cell wall biosynthesis, and inactivation of lipopolysaccharides in bacteria like *Staphylococcus aureus, Escherichia coli*, and *Pseudomonas aeruginosa* by specific polyphenol compounds, including catechin, quercetin, epigallocatechin gallate, myricetin, ferulic acid, gallic acid, proanthocyanidins, tannin, and kaempferol-3-rutinoside.

**Table 3** shows the various polyphenol sources, kinds, antimicrobial properties, and their mechanisms of action. **Figure 4** illustrates the antimicrobial mechanisms of polyphenols, illustrating the disruption of microbial cell structures (lipopolysaccharide cell wall, peptidoglycan cell wall, phospholipid bilayers, cell membrane proteins) and the impairment of essential cellular functions (inhibition of DNA gyrase and RNA synthesis, pore formation causing leakage, damage to membrane lipid bilayers, inhibition of enzyme activity, disruption of cell wall biosynthesis, and inactivation of lipopolysaccharide) in bacteria such as *S. aureus*, *E. coli*, and *P. aeruginosa* by specific polyphenol compounds (catechin, quercetin, EGCG, myricetin, ferulic acid, gallic acid, proanthocyanidins, tannin, and kaempferol-3-rutinoside).

### 5.4 Anti-diabetic activity

Natural products play a significant role in promoting human health (365, 366). Plants have long been used in various cultures to treat diseases and disorders (367, 368). Accordingly, research continues to explore plant-derived compounds for managing type 2 diabetes mellitus, a metabolic disorder increasingly prevalent due to modern lifestyle changes (369, 370). Type 2 diabetes mellitus is characterized by chronic hyperglycemia resulting from insulin resistance, amyloid deposition, pancreatic β-cell dysfunction, and impaired glucose regulation (371, 372).

Current studies indicate that insulin regulation involves several mechanisms, including pancreatic cell protection, modulation of cell proliferation and apoptosis, oxidative stress reduction, insulin signaling activation, increased insulin secretion, inhibition of glucose uptake, gut microbiome regulation, and attenuation of inflammatory responses (16, 373). Therefore, dietary polyphenols hold the potential for managing type 2 diabetes mellitus (374, 375). Additionally, compounds such as resveratrol, curcumin, and quercetin have shown that they can lower oxidative stress and inflammation by modulating key insulin-related signaling pathways (14, 376). Numerous studies have reported the anti-diabetic effects of tea polyphenols in experimental diabetes models, demonstrating their ability to lower blood glucose levels, improve insulin sensitivity, and reduce oxidative stress and inflammation associated with type 2 diabetes mellitus (377, 378).

Sabu et al. (377) found that administration of 500 mg/kg green tea polyphenols significantly inhibited the increase in serum glucose levels at 60 min. Similarly, polyphenols extracted from spicate eugenia (*Syzygium zeylanicum* L.) exhibited anti-diabetic influences in 2.5–3-month-old diabetic zebrafish subjected to overfeeding and hyperglycemic conditions. The findings suggest these polyphenols may regulate genes involved in lipid and glucose metabolism and influence glucose absorption and utilization, contributing to the normalization of fasting blood glucose levels (379). Animal studies have also demonstrated the anti-diabetic effects of flax (*Linum usitatissimum*) in 8–12-week-old female rats, with consistent reductions in blood glucose levels and body weight (380). Histological analyses revealed partial improvement in pancreatic, hepatic, and renal tissues following treatment with the plant extract (380).

Zuo et al. (381) investigated the anti-diabetic properties of *Phaseolus vulgaris* L. in 5–6-week-old male rats. In this study, type 2 diabetes mellitus rats were fed either a high-fat diet or a standard diet with detailed macronutrient compositions. The results showed that *P. vulgaris* L. could regulate blood glucose and cholesterol levels, reduce insulin resistance, and increase gut short-chain fatty acid production, thereby mitigating pancreatic and hepatic damage and restoring intestinal microbiota balance (381). Another study assessed the anti-diabetic properties of yellow and green papaya (*Carica papaya*), revealing lipid-lowering activity and enhanced hepatic glucose metabolism, suggesting its therapeutic potential in diabetes management (382).

Similarly, Pieczykolan et al. (383) revealed that *Aerva lanata* L. has been shown to possess anti-diabetic, antioxidant, and anti-inflammatory properties via inhibition of α-amylase and α-glucosidase, enzymes associated with glucose metabolism. Further investigations have explored the anti-diabetic potential of ethanolic propolis extracts under *in vitro* and *in vivo* conditions (384). In one experiment, diabetic rats were administered a 0.5 mL/100 g dose of either 15% or 30% propolis extract for 4 weeks, resulting in significant blood glucose reduction (384). A separate study investigated the therapeutic effects of vinegar extract from *Zhenjiang* aromatic vinegar in diabetic mice. The extract improved body weight, lowered blood glucose, enhanced glucose and insulin tolerance, and reduced liver inflammation. These effects were partly attributed to the modulation of the gut microbiota and short-chain fatty acid levels, indicating a potential role in diabetes therapy (385).

Vaithiyalingam et al. (386) investigated the pharmacokinetic characteristics of curcumin from *Curcuma longa*, highlighting its strong ligand-binding interactions with key protein targets, including α-amylase, α-glucosidase, DPP-4, PPAR, and SGLT-2. These findings position curcumin as a promising candidate for diabetes treatment. Jahan et al. (387) examined the functional potential of haustoria from coconut (*Cocos nucifera*) and palmyra palm (*Borassus flabellifer*) for anti-diabetic applications. *B. flabellifer* demonstrated superior antioxidant capacity through DPPH and H_2_O_2_ scavenging and lipid peroxidation inhibition compared to *C. nucifera*. Additionally, α-amylase and α-glucosidase inhibition assays showed greater enzyme inhibitory activity in *B. flabellifer* (387).

The anti-diabetic efficacy of diverse polyphenols has been demonstrated in both *in vivo* and *in vitro* studies, supporting their potential as therapeutic agents (388, 389). However, despite promising findings, current research remains insufficient, and further investigations are needed to validate these compounds for future clinical applications.

**Figure 5:**
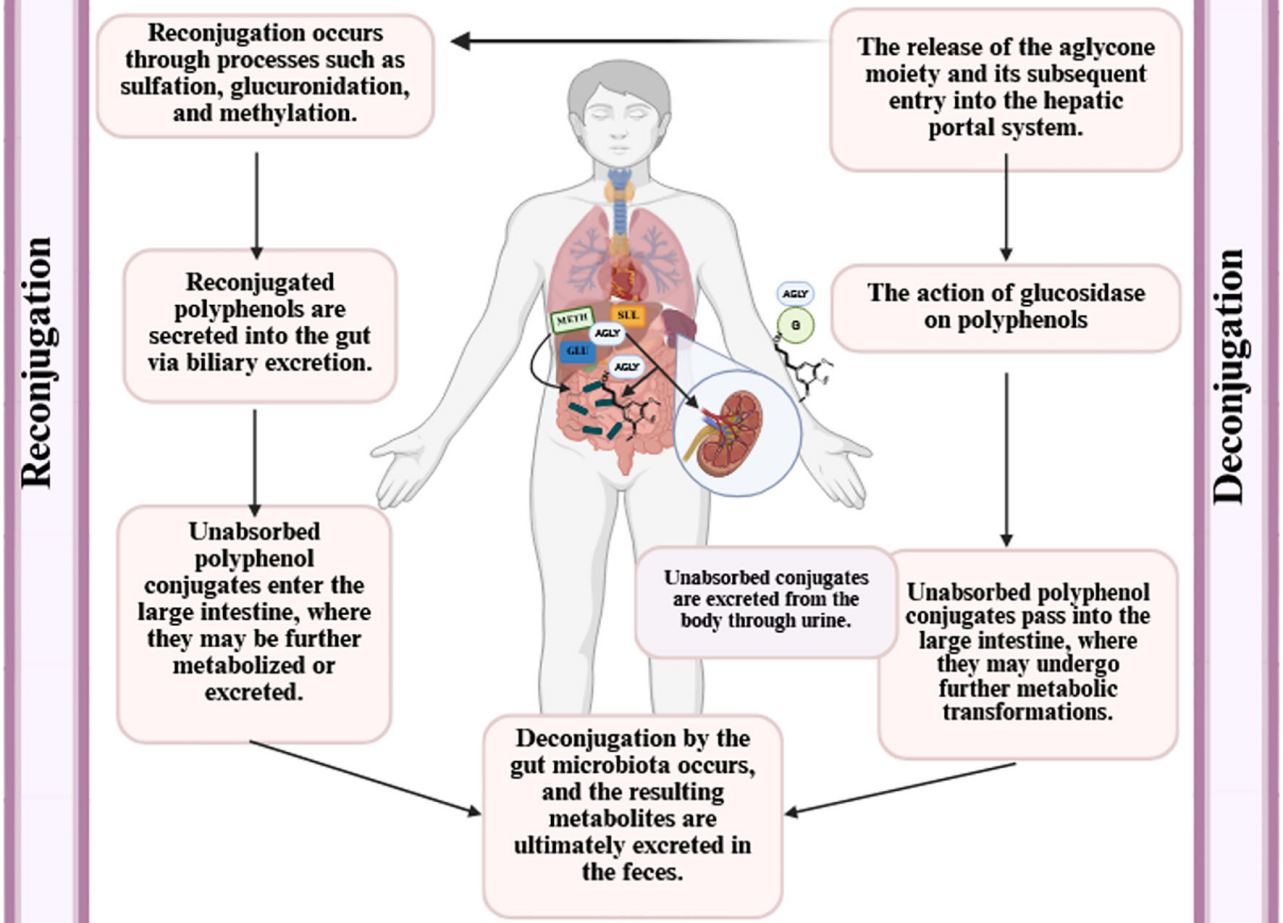
Antidiabetic mechanism of polyphenols in the human body, illustrating deconjugation in the gastrointestinal tract, absorption, hepatic portal circulation, reconjugation in the liver, biliary excretion, renal excretion, and microbial deconjugation in the colon, resulting in fecal excretion.

**Figure 5** illustrates the antidiabetic mechanism of polyphenols in the human body, showing deconjugation in the gastrointestinal tract, absorption, hepatic portal circulation, reconjugation in the liver, biliary excretion, renal excretion, and microbial deconjugation in the colon, ultimately leading to fecal excretion.

### 5.5 Application in skin and hair health

The skin, the body’s largest organ, acts as a dynamic interface with the environment, playing essential roles in protection against UV radiation, pathogens, and extreme temperatures (390, 391). Its constant exposure to environmental stressors, combined with its complex functions, contributes to the development of various dermatological conditions (392). Similarly, hair follicles—extensions of the epidermis—are influenced by both internal and external factors. Hair follows a cyclical growth pattern encompassing the anagen, catagen, and telogen phases. Disruptions in this cycle can lead to hair thinning or loss, adversely affecting an individual’s psychosocial well-being, self-esteem, and mental health, often leading to social anxiety and depression (392).

A range of factors—genetic predisposition, hormonal imbalances, infections, stress, and psychological disorders—contribute to both skin and hair disorders. While conventional pharmaceutical and treatments are available, many synthetic drugs pose limitations or adverse effects, fueling growing interest in natural alternatives (391). Among these, plant-derived polyphenols have garnered attention for their broad therapeutic potential, including anti-inflammatory, antioxidant, anti-aging, anti-carcinogenic, antimicrobial, and depigmenting properties (393).

Several studies have highlighted the potential of polyphenols in dermatological applications. For example, grape seed extract, applied to normal human melanocyte and dermal fibroblast cells, was shown to enhance skin youthfulness by stimulating collagen and elastin production (133). It also reduced UVB-induced inflammation and DNA damage, while promoting skin hydration and reducing melanin production—key factors in diminishing wrinkle formation (133).

Similarly, *Caralluma europaea* extracts demonstrated both anti-tumor and wound-healing effects. These extracts inhibited leukemia and hepatocellular carcinoma cell lines *in vitro*, while topical application in rats accelerated wound healing (394). Ethanolic extracts of *Acacia nilotica* showed potent free radical scavenging activity due to their hydroxyl group content, indicating their potential as natural antioxidants (393).

In a clinical trial by Montenegro et al. (395), the topical application of resveratrol-loaded lipid nanocarriers significantly improved skin hydration, emphasizing the potential of lipid-based delivery systems in skincare. Other studies showed that combining polyphenols with sunscreen components provided synergistic UV protection (377). Notably, naringenin-loaded NPs exhibited superior antioxidant activity and sustained skin retention compared to the native compounds (395).

Curcumin-loaded nanocubosomal hydrogels were found to reduce signs of skin irritation (e.g., erythema and edema) in rats and displayed enhanced antibacterial activity against *E. coli* (396). Likewise, a hydrophilic extract of *Rhus coriaria* promoted collagen production, accelerated wound healing, and showed antimicrobial activity against several pathogenic bacteria (397). A liposome-based extract from *Hibiscus sabdariffa* L. calyx showed no irritation in rabbit skin models and was effective as an anti-aging skincare product due to its antioxidative properties (398). Polyphenols from *Malpighia emarginata* DC also demonstrated skin-lightening effects by reducing UVB-induced pigmentation and melanin synthesis (399). Similarly, strawberry extracts protected against UVA-induced skin damage by reducing ROS and inflammatory markers (400).

Other findings revealed that *Penthorum chinense* extracts possess anti-aging and moisturizing properties (383), and green tea polyphenols exhibit anti-inflammatory effects against acne vulgaris (401). In another study, *Coffee arabica* L. hydrogels—especially those from green beans—promoted skin regeneration and reduced oxidative stress in wound areas (402). Overall, polyphenols offer significant potential in promoting skin and hair health (402). However, more comprehensive studies are needed to optimize their application and explore their full therapeutic potential in dermatology.

### 5.6 Neuroprotective effect

Polyphenols are increasingly recognized for their neuroprotective properties, primarily attributed to their potent antioxidant and anti-inflammatory activities (377, 403). These compounds can neutralize free radicals and reduce oxidative stress, mechanisms implicated in the pathogenesis of several neurodegenerative diseases, including Alzheimer’s disease and Parkinson’s disease (404, 405).

Curcumin, a polyphenol derived from *C. longa*, has demonstrated anti-inflammatory, antioxidant, and anti-amyloid effects in various Alzheimer’s disease models (388). For instance, curcumin-loaded lipid-core nanocapsules mitigated Aβ-induced behavioral changes and synaptotoxicity in rats. The purified polyphenols from pomace significantly reduced paralysis in *Caenorhabditis elegans* Alzheimer’s disease and showed antioxidant effects compared to non-purified forms (388).

In Alzheimer’s disease, the accumulation of β-amyloid (Aβ) peptides and tau protein aggregates disrupts neuronal function (406). Blends of polyphenols—including resveratrol and grape juice—have been shown to reduce amyloid neuropathology and improve cognitive deficits in animal models (406). Resveratrol, in particular, activates the *Sirt1* gene, enhances glutathione and superoxide dismutase levels, and reduces oxidative stress (407). Grape leaf polyphenols also exhibited neurotrophic, anti-inflammatory, and antioxidant effects in aluminum chloride-induced Alzheimer’s disease rat models, suggesting a potential therapeutic role (408).

Parkinson’s disease, characterized by the degeneration of dopaminergic neurons and the aggregation of α-synuclein, polyphenols again show promise (409). EGCG from green tea has been shown to inhibit α-synuclein aggregation and prevent mitochondrial dysfunction (410, 411). A unique polyphenol-micronutrient blend, A5+, was found to block apoptotic pathways, reduce oxidative stress, and suppress pro-inflammatory cytokines in Parkinson’s disease models (387). In addition, nanosheet polyphenolic fractions from propolis demonstrated enhanced antioxidant effects *in vitro* and *in vivo* (412). Olive-derived polyphenols improved locomotor ability and lifespan in *C. elegans* Parkinson’s disease models (405). In Huntington’s disease, caused by polyglutamine expansions in the Huntingtin protein, curcumin reduced photoreceptor degradation and motor impairment in *Drosophila* models (413).

Despite promising evidence, challenges remain (414, 415). The mechanisms underlying the neuroprotective effects of polyphenols are not fully understood, and issues with their bioavailability persist (416, 417). Future research is essential to elucidate these mechanisms, improve delivery systems, and determine effective therapeutic dosages for preventing and managing neurodegenerative diseases.

### 5.7 Anti-tumor and anti-cancer activity

Researchers have long been interested in exploring the anti-tumor and anti-cancer potential of polyphenols (418, 419). These natural chemicals exhibit chemo-preventive benefits against multiple cancer types, as shown in **Table 4** (465, 466). Research suggests that polyphenols may significantly limit tumor growth and prevent cancer formation due to their anti-inflammatory, antioxidant, and antiproliferative effects (467, 468). Multiple methodologies exist to evaluate the antiproliferative, and anti-cancer properties of polyphenols (469, 470). One strategy entails performing *in vitro* research using cancer cell lines (471, 472). Researchers can examine the effects of polyphenols derived from various sources or using different quantities on tumor cells, and their effects on cellular growth and proliferation (473, 474).

**Table 4:** Sources, types, anti-cancer and antitumor properties, and the mechanism of action of polyphenols.

Polyphenol sources	Polyphenols	Anti-cancer and anti-tumor activities	Mode of action	References
Green tea	Epigallocatechin gallate (EGCG)	Inhibits cancer cell proliferation and induces apoptosis in various cancer cell lines	Modulates signaling pathways such as MAPK, inhibits angiogenesis, and induces epigenetic changes	(230, 420)
Black tea	Theaflavin-3,3’-digallate (TFDG)	Exhibits antioxidant properties and inhibits cancer cell growth	Scavenges reactive oxygen species and inhibits angiogenesis by reducing VEGF production	(421, 422)
Red grape	Resveratrol	Suppresses tumor initiation, promotion, and progression	Modulates gene expression related to cell proliferation and apoptosis, and inhibits angiogenesis	(423, 424)
Turmeric	Curcumin	Inhibits the growth of various cancer cells and induces apoptosis	Modulates multiple cell signaling pathways, including NF-κB and STAT3, and alters epigenetic regulation	(425, 426)
Soybean	Genistein	Inhibits cancer cell growth and metastasis	Modulates estrogen receptor signaling and inhibits tyrosine kinase activity	(286, 427)
Apple	Phloretin	Inhibits the proliferation of cancer cells and induces apoptosis	Inhibits glucose transporters and modulates cell cycle regulators	(428, 429)
Berry	Anthocyanins	Suppresses the growth of various cancer cells and induces apoptosis	Inhibits oxidative stress and modulates signaling pathways such as PI3K/Akt	(430, 431)
Pomegranate	Ellagic acid	Inhibits cancer cell proliferation and induces apoptosis	Modulates cell cycle regulators and inhibits angiogenesis	(432, 433)
Olive oil	Hydroxytyrosol	Inhibits the proliferation of cancer cells and induces apoptosis	Scavenges reactive oxygen species and modulates signaling pathways	(434, 435)
Citrus fruit	Hesperidin	Inhibits cancer cell growth and induces apoptosis	Modulates signaling pathways such as MAPK and inhibits angiogenesis	(436, 437)
Garlic	Quercetin	Inhibits the proliferation of cancer cells and induces apoptosis	Modulates signaling pathways and inhibits oxidative stress	(438, 439)
Broccoli	Sulforaphane	Inhibits cancer cell growth and induces apoptosis	Modulates epigenetic regulation and inhibits histone deacetylase activity	(440, 441)
Tomato	Lycopene	Inhibits the proliferation of cancer cells and induces apoptosis	Modulates signaling pathways and inhibits oxidative stress	(213, 442)
Chili pepper	Capsaicin	Inhibits the growth of various cancer cells and induces apoptosis	Modulates signaling pathways such as NF-κB and induces oxidative stress	(443, 444)
Ginger	6-Gingerol	Inhibits the proliferation of cancer cells and induces apoptosis	Modulates signaling pathways and inhibits oxidative stress	(445, 446)
Cranberry	Proanthocyanidins	Inhibit the growth of various cancer cells and induce apoptosis	Inhibit oxidative stress and modulate signaling pathways	(447, 448)
Spinach	Lutein	Inhibits the proliferation of cancer cells and induces apoptosis	Scavenges reactive oxygen species and modulates signaling pathways	(449, 450)
Carrot	Beta-carotene	Inhibits cancer cell growth and induces apoptosis	Scavenges reactive oxygen species and modulates gene expression	(451, 452)
Flaxseed	Secoisolariciresinol diglucoside (SDG)	Inhibits the proliferation of cancer cells and induces apoptosis	Modulates estrogen receptor signaling and inhibits oxidative stress	(453, 454)
Cheery	Cyanidin	Inhibits the growth of various cancer cells and induces apoptosis	Inhibits oxidative stress and modulates signaling pathways	(455, 456)
Peanut	Pterostilbene	Inhibits the proliferation of cancer cells and induces apoptosis	Modulates signaling pathways and inhibits oxidative stress	(457, 458)
Parsley	Apigenin	Inhibits cancer cell growth and induces apoptosis	Modulates signaling pathways such as NF-κB and inhibits angiogenesis	(459, 460)
Thyme	Luteolin	Inhibits the proliferation of cancer cells and induces apoptosis	Modulates signaling pathways and inhibits oxidative stress	(461, 462)
Rosemary	Carnosic acid	Inhibits the growth of various cancer cells and induces apoptosis	Modulates signaling pathways and inhibits oxidative stress	(463, 464)

Zhang et al. (456) indicated that *Cerasus humilis* fruit, recognized for its high polyphenol content, demonstrated considerable inhibitory effects on hepatic, colon, and stomach tumor cells. A modern experiment demonstrated that the phenolic component of *Cerasus europaea* extracts displayed ‘anti-tumor’ potential versus human leukemia (K562 and HL60) and hepatic tumor (Huh-7) cell lines (394). Yi et al. (475) showed that the purified polyphenols possess antiproliferative properties on distinct cancer cell lines in the human colon tumor stem cell line (LOVO cell line), the pure polyphenols acquired in this research may be utilized to manufacture functional meals.

Furthermore, Yi et al. (476) conducted a study demonstrating that isolated polyphenols from *Pinus koraiensis* pinecones have an anti-cancer impact on colon cancer cells via stimulating death via caspase activation. Huang et al. (477) indicated that the extracted polyphenols from the bark of *P. koraiensis* have a significant inhibitory effect on colon cancer cells via augmenting the quantity of apoptotic cells (477). The investigation examined the use of several polyphenols to mitigate the side impacts of tumor, beside their direct impacts on tumor cells (478–480).

Another method to examine these impacts is by performing *in vivo* experiments with animal models (481, 482). These investigations often entail observing cancer progression in affected animals via the supply of polyphenols using diverse approaches (482, 483). Extensive research may include both *in vitro* and *in vivo* trials (484, 485).

These findings clarify the molecular mechanisms influenced by polyphenols (472, 486, 487). The fundamental purpose of these investigations is to study the impacts of polyphenols on apoptosis, angiogenesis, cell cycle regulation, and metastasis (488, 489). Wu et al. (488) revealed a notable reduction in cell viability corresponding to elevated dosages of polyphenols derived from *Hippophae rhamnoides* labeled as HPs60, showing an inactivation impact of HPs60 on tumor cell proliferation. The modification of miRNA expression patterns resulting from HPs60 therapy influenced the alterations in cell viability through modulating cell cycle progression as well as apoptosis (488). *In vivo* investigations on mice indicated no discernible poisonousness throughout HPs60 therapy, as demonstrated by the lack of substantial changes in body weight across the groups (488). In contrast, there was a notable decrease in cancer volume following HPs60 therapy relative to the control, demonstrating its anti-tumor efficacy in inhibiting cancer growth *in vivo*. Moreover, HPs60 therapy was demonstrated to influence the expression of microRNAs (miRNAs) in cancer-bearing animals (488).

Moreover, the group receiving the blueberry anthocyanin and crude polyphenol extract demonstrates the most significant cancer suppressor impacts, presumably due to synergistic interactions between the components (490, 491). Furthermore, the extract improved the overall health of mice by augmenting cellular immunological action, enhancing antioxidant enzymatic activities, and diminishing lipid peroxidation (492, 493). To conclude, various studies indicate that polyphenols might substantially affect cancer prevention, influencing disease progression and perhaps improving treatment methods such as radiotherapy and chemotherapy (483, 492, 493). **Table 4** delineates the sources, classifications, anti-cancer and antitumor characteristics, as well as the mechanisms of action of polyphenols.

### 5.8 Other effects

The influence of polyphenols on health encompasses multiple aspects (494, 495). A study approved by de Jesús Romero‐Prado et al. (496) indicates a significant reduction in both systolic as well as diastolic blood pressure, as well as decreases in whole cholesterol, LDL cholesterol, as well as triglyceride levels after the inclusion of dietary flavonoids. The incorporation of flavonoids into pharmacological antihypertensive treatment demonstrates supplementary advantages for blood pressure, lipid profile, leptin levels, obesity, and inflammation (496). Polyphenols contribute to reducing obesity through a variety of interrelated mechanisms. They can inhibit adipogenesis by regulating key signaling pathways and transcription factors such as PPARγ and C/EBPα, thereby limiting the formation and differentiation of new adipocytes (497). Polyphenols also promote the browning of white adipose tissue and enhance thermogenesis, which increases energy expenditure and fat burning (497). Additionally, these compounds stimulate β-oxidation of fatty acids, promote lipolysis, and suppress lipogenesis, collectively improving lipid metabolism and reducing fat accumulation (498).

Moreover, a notable reduction in levels of C-reactive protein was seen, suggesting a possible role in reducing the hazard of cardiovascular disorders. Bogolitsyn et al. (276) observed that polyphenols elevated the quantity of sticky leukocytes in the bloodstream of both leukemia cases and healthy people. Moreover, leukocytes from leukemia cases exhibited a reduced propensity to attach to surfaces relative to those from healthy people, suggesting that algal polyphenols regulated the adhesive activities of leukocytes in a dose-reliant methods. Moreover, polyphenols augmented the adhesion and contact capabilities of cells by stimulating defensive mechanisms against malignant cells (276).

**Figure 6:**
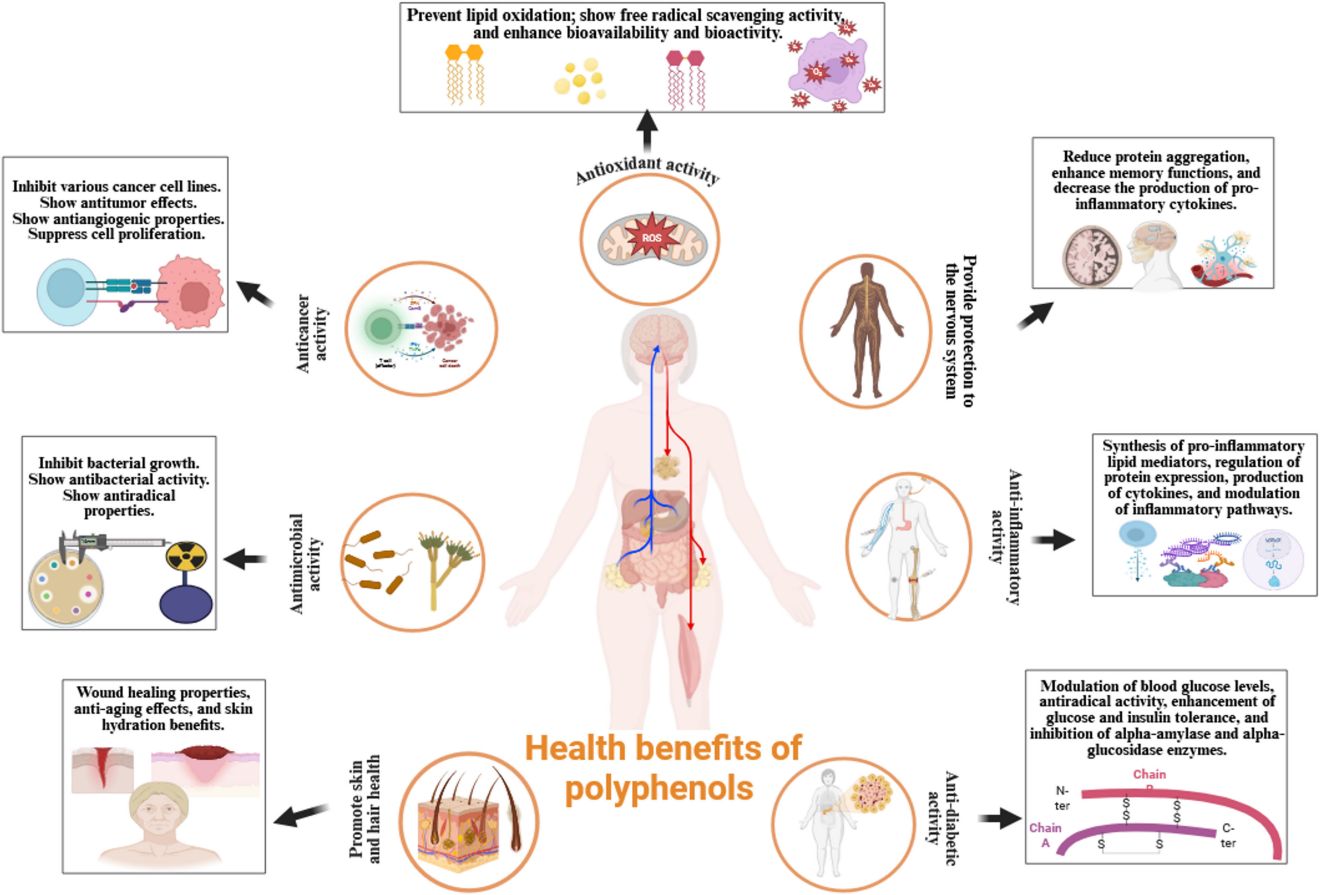
The numerous health advantages of polyphenols in the human body demonstrate their antioxidant, anti-cancer, antibacterial, dermatological, neuroprotective, anti-inflammatory, and anti-diabetic properties, as well as their modes of action at both cellular and systemic levels.

**Figure 6** outlines the various health benefits of polyphenols within the human body, highlighting their antioxidant, anticancer, antibacterial, dermatological, neuroprotective, anti-inflammatory, and anti-diabetic properties, as well as their modes of action at both cellular and systemic levels.

## 6 Polyphenols and nutritional aspects

Research indicates that the intake of polyphenol-rich foods can promote health, chiefly owing to their antioxidant, anti-inflammatory, anticarcinogenic, and several other qualities (499, 500). Moreover, these qualities are believed to support intestinal health by fostering the proliferation of good bacteria (495). These attributes promote the intake of foods abundant in polyphenols (501), yielding numerous beneficial consequences (502, 503).

**Figure 7:**
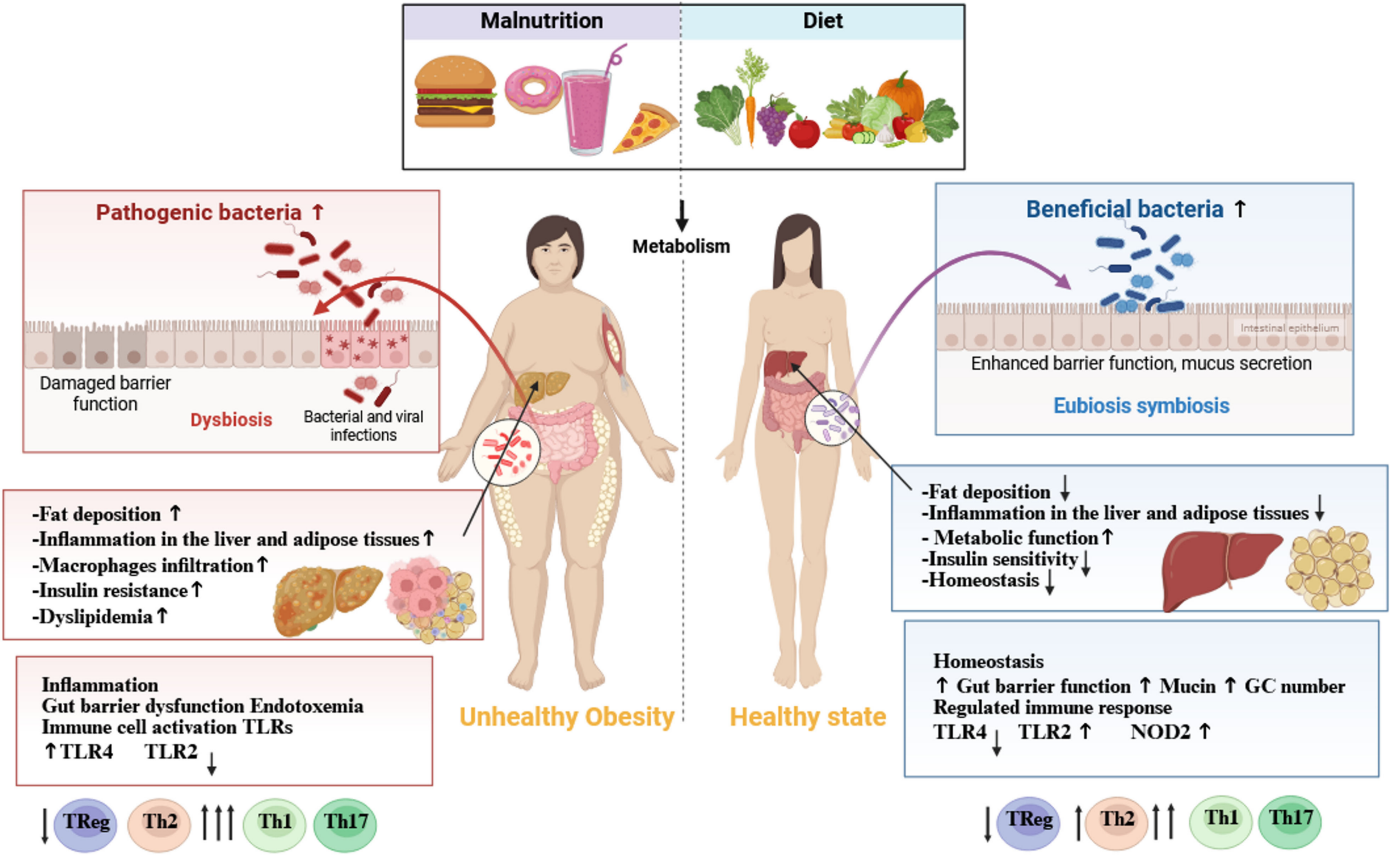
The effects of malnutrition (high-fat, high-sugar diet) compared to a healthy diet (fruits and vegetables) on metabolism, gut microbiota composition (dysbiosis vs. eubiosis), intestinal barrier integrity, systemic inflammation, insulin resistance, dyslipidemia, adipose tissue accumulation, and immune cell responses (TLR4, TLR2, Treg, Th1, Th2, Th17) resulting in detrimental obesity as opposed to a healthy metabolic state.

**Figure 7** illustrates the impact of malnutrition (high-fat, high-sugar diet) in contrast to a nutritious diet (fruits and vegetables) on metabolism, gut microbiota composition (dysbiosis vs. eubiosis), intestinal barrier integrity, systemic inflammation, insulin resistance, dyslipidemia, adipose tissue accumulation, and immune cell responses (TLR4, TLR2, Treg, Th1, Th2, Th17), culminating in adverse obesity rather than a healthy metabolic condition.

### 6.1 Role of nutrition and gut microbiota in maternal and infant health

The early stage of life is crucial for the growth of the infant’s gastrointestinal microbiome. The maturation of the gastrointestinal microbiome throughout infancy and early childhood can affect health and the likelihood of disorders in later life (504). Alterations in the gut microbiota throughout this time precipitate the onset of chronic disorders such as asthma, allergies, and obesity in both adulthood and childhood (505, 506). Studies indicate that human milk plays a crucial role in establishing an infant’s gut microbiome and serves as a significant source. Consequently, the mother’s breastfeeding practices safeguard the infant versus gastrointestinal as well as respiratory pathogens, while also mitigating the dangers of inflammatory deterioration (507).

Maternal microbial components are believed to be transmitted to the newborn via human milk, along with the transfer of non-microbial molecules (508). Consequently, research elucidating the connection between nutrition and gastrointestinal microbiome in adults has sought to demonstrate a correlation with postpartum mothers (508, 509). The dietary habits of postpartum mothers, modifications to these habits, and the variety of food types ingested influence the mother’s microbiota, thus altering the human milk microbiota. This may subsequently influence the gut flora of the newborns (508).

Polyphenols, chemicals essential for plant defense, are prevalent in individual foods. Polyphenols obtained from diverse food sources exert advantageous impacts on various metabolic problems, cognitive decline, and offer protection against conditions such as tumors and aging (510). Among these, beneficial effects such as antioxidant and anti-inflammatory qualities, as well as modulation of hormonal and mitochondrial functions, polyphenols may enhance mother milk production, as well as nursing efficacy (510). The nature and composition of the mother’s food are crucial for the infant’s health, both throughout pregnancy and throughout the breastfeeding period, including the time before and after this phase (510). Consequently, a Mediterranean diet-style regimen, abundant in polyphenols and fiber, enhances the mother’s nutritional profile and is beneficial for maternal and newborn health (511). Limited research with dairy animals, like goats and cows, have shown that polyphenols from fenugreek (*Trigonella foenum*-graecum L.) enhance milk output and improve the quality and content of milk lipid (511, 512). Fenugreek is predominantly utilized to enhance the quality of milk production in postpartum mothers (513).

Fenugreek encompass several polyphenols, including quercetin, isovitexin, rutin, vitexin, diosgenin, and saponins (514). Additionally, other research indicates that fenugreek significantly enhances milk flow, yield, oxytocin expression, and lipid level in pregnant rats (515, 516). Scholars have shown that *Moringa oleifera*, which contains various flavonoids such as kaempferol, myricetin, quercetin, and phenolic acid, influences milk level and enhances the macronutrient content, involving protein and lipid, in dairy animals (517, 518). In contrast, Olvera-Aguirre et al. (519) indicated no impact on milk supply or quality on dairy animals utilizing the same herb.

*In vitro* studies suggest that extracts or leaves of the *M. oleifera* can diminish ROS, and enhance glutathione levels and casein gene expression in bovine mammary cells. *M. oleifera* exhibits a preventive function versus induced ROS *in vitro* (520, 521). Additionally, various herbal formulations, such as fenugreek*, Sauropus androgynus*, and *M. oleifera*, were evaluated for their lactation-enhancing effects on lactating rats (522, 523). The study’s findings indicated an increase in milk output. Furthermore, several animal experiments, including female rats and Balb/c mice, demonstrated the lactation hormone-stimulating effect of milk thistle and *S. androgynus* (524, 525). These research findings indicate that the prolactin hormone’s expression, linked to enhanced breast milk production in postpartum women, and the oxytocin hormone, recognized as the milk-ejecting hormone, were elevated (526, 527).

Sani et al. (528) investigated the impact of the polyphenol resveratrol from the *Launaea taraxacifolia* on milk yield and serum levels of oxytocin and prolactin in rats. The study’s results demonstrate that resveratrol may enhance milk production and elevate prolactin and serum oxytocin levels (528). One study indicated that quercetin polyphenols enhance prolactin formation in the pituitary gland, while another study showed that the same polyphenol from the *Ligustrum lucidum* could diminish the inflammation of the mammary gland (529, 530). Zhao et al. (531) showed that orange peel extract increases milk output among dairy animals, and Ceballos-Sánchez et al. (511) demonstrated that this fiber and polyphenol-rich diet supplemented exerted a trophic impact on both the pregnant rats and their newborns. Nevertheless, additional research is required to clarify the mechanisms (511).

### 6.2 Role of polyphenols from childhood to the elderly

The intake of polyphenol-rich foods is essential for people across all age groups, including children, adults, and the elderly. Integrating polyphenol-rich meals can improve growth and development in children and adolescents (532). Moreover, polyphenols may be ingested to improve cognitive action and overall health, especially in adults and the elderly (533, 534).

Moreover, incorporating polyphenols into the diet of adults and the elderly can enhance overall health, diminish the hazard of chronic disorders, and promote cardiovascular well-being (535, 536). Ziauddeen et al. (532) analyzed documents from the National Diet and Nutrition Survey Rolling Programme (NDNS RP) 2008–2014 to evaluate polyphenol consumption among the UK population. The study results suggested that polyphenol consumption escalated with age, with a more pronounced increase observed in male subjects. In children, the principal sources of polyphenols were potatoes, legumes, fruit juice, and tea. The primary sources of polyphenols for adults include chocolate, tea, fruits, wine, and vegetables (532).

Corbo et al. (537) indicated that sweet cherry polyphenol extracts suppressed spontaneous osteoclastogenesis in obese youngsters by diminishing the development of multinucleated TRAP+ osteoclasts in peripheral blood mononuclear cell cultures (537). Moreover, the polyphenol extracts diminished the capacity of peripheral blood mononuclear cells to create extensive resorption zones on calcium phosphate film-coated Millenium slides, consequently impeding the bone resorption functions of osteoclasts and reducing TNFα mRNA levels (537). Conversely, the evaluation of polyphenol extracts on cell viability in peripheral blood mononuclear cell cultures, conducted via the MTT assay, revealed that these extracts were non-toxic and promoted the preservation of healthy cells (537). The research findings indicate that sweet cherry extracts abundant in polyphenols can aid in the prevention and/or enhancement of bone health issues related to obesity (537).

A separate study by Whyte and Williams (538) noted that blueberry anthocyanins positively influenced some memory actions in youngsters, however, this impact did not encompass all cognitive domains. Moreover, participants who ingested the blueberry beverage exhibited superior performance relative to people who took a placebo, especially in long-delay recall tasks of children in 10-year-olds (538). A study including 400 children aged 4 to 12 aimed to examine the correlation between dietary polyphenol intake and the risk of attention deficit hyperactivity disorder (539). Polyphenols may offer protection against attention deficit hyperactivity disorder by altering membrane fluidity and adrenergic receptors, demonstrating antioxidant properties, inducing vasodilation, and regulating catecholamine metabolism (539).

Mengfa et al. (540) examined the correlation between polyphenol consumption and the incidence of type 2 diabetes. The results indicated that a higher consumption of polyphenols correlated with a diminished risk of type 2 diabetes. Guo et al. (541) indicated that polyphenol consumption may decrease obesity risk in elderly adults with elevated cardiovascular risk. Guglielmetti et al. (542) indicated that a diet high in polyphenols positively influenced gut permeability in ageing, leading to reduced serum zonulin levels. Decreases were noted in inflammatory markers like IL-6, C-reactive protein, and TNF-α, ROS indicators involving DNA injury, and measures of vascular action. Moreover, polyphenol-rich diets help preserve metabolomic profiles and microbiome equilibrium in the elderly (542).

### 6.3 Role of polyphenols on athlete health

One advantageous outcome of addressing the athlete’s nutritional requirements is enhancing athletic performance. Environmental, endocrine, muscular fiber relationships, athletic objectives, dietary, and genetic factors create individual variances that may also influence athletic performance (543). Genetic and dietary combinations can influence nutritional availability and bodily systems associated with athletic performance (544). The amount and composition of macronutrients, lipids, carbohydrates, and proteins in a person’s dietary regimen significantly influence athletes’ muscular functions and performance (544). Recent evidence indicates that the kind and amount of protein are essential for muscle hypertrophy and athletic performance, with individual differences in protein consumption and amino acid absorption-metabolism associated with both protein quantity and quality, as well as genetic variances between persons (545).

Genetic differences might change the quantity of bioactive peptides obtained from protein resources, hence influencing muscle function and development (546). Consequently, daily nutritional guidance includes tailored nutritional suggestions for each athlete throughout training and pre-, intra-, and post-competition periods (546). Alongside these macronutrients, it is advisable to daily ingest foods abundant in manganese, butyrate, omega-3, and polyphenols, and to contemplate incorporating supplements such as antioxidants and anti-inflammatories (546). Moreover, nourishment supplies energy to the body and helps maintain physiological equilibrium. Furthermore, diet is crucial in enhancing the body’s reaction to exercise-induced stress (547).

Consequently, an athlete must regulate the homeostasis of oxidative stress while training. Oxidative stress, resulting from the generation of ROS, can lead to inflammation and cellular destruction and impede muscle recovery if it coincides with training adaptations (548). Antioxidant supplementation during exercise influences athletic performance, but mitochondrial adenosine triphosphate generation is not entirely effective, forming superoxide radicals. The increased oxygen consumption leads to a higher generation of superoxide radicals that require neutralization (548). Muscle injury results in excessive free radical production, hindering recovery, and the body's intrinsic systems for eliminating these radical species are inadequate (548).

Plant-based diets are garnering interest in contemporary sports nutrition because of their substantial nutrients of bioactive compounds (549). Polyphenols offer several benefits for athletes, including anti-inflammatory, antioxidant, and antimicrobial characteristics, promoting overall wellness (550). These advantages have linked some polyphenols, such as resveratrol, quercetin, and curcumin, to muscle health (551). Numerous studies on sports nutrition and polyphenols are now underway. Many of these studies encompass the significant impacts of polyphenolic materials on post-exercise muscle destruction as well as their influence on enhancing physical performance (552). Polyphenol compounds have been investigated under various situations employing diverse supplementation regimens for differing periods and dosages (503). Polyphenols, extensively researched for their numerous beneficial effects. Consequently, diets rich in polyphenols were examined to mitigate oxidative stress induced by physical performance (553, 554).

Additionally, the impact of quercetin polyphenol supply on athletic performance was examined. Quercetin, a flavonoid polyphenol, plays a crucial function in muscle remodeling by inhibiting muscle loss by controlling protein catabolism and promoting muscular anabolism via increased phosphorylation (555). A study of top cyclists revealed enhancements in aerobic performance among athletes consuming 1200 mg of the supplement daily for six weeks (556). Sgrò et al. (557) indicated that the group administered 1 g of quercetin daily for two weeks exhibited reduced plasma markers of eccentric muscle injury relative to the placebo group. This indicates that quercetin facilitates the regeneration of muscle injury (557). A study by Martin-Rincon et al. (558), including 24 female and 33 male active athletes, attempted to assess their conditions following long-distance running performances of five and ten kilometers (558). The results indicated that the blend of Zynamite and quercetin mitigates muscle discomfort and injury while expediting the therapy of muscular performance. The advantages of quercetin supply are believed to be enhanced with elevated levels (558). Additionally, polyphenols such as resveratrol, which are predominantly found in red wine and grape skin, can stimulate anabolic muscle metabolism by augmenting signaling pathway components (545). de Sousa et al. (559) indicated that the grape juice supplement enhanced the athletes’ endurance times.

A separate study by de Lima Tavares Toscano et al. (560) observed that one dose of purple grape juice demonstrated an ergogenic impact in recreational runners via prolonging duration to exhaustion throughout running and enhancing antioxidant (560). In addition to this, animal research are also incorporated in the literature (561, 562). Nonetheless, the limited sample sizes in resveratrol studies and the application of numerous unspecified supplement dosages hinder the establishment of a definitive safety and efficacy range for this supplement in athletes; thus, further research is required (562).

Besides the advantages of resveratrol administration for athletes, it may also regulate glucose and insulin sensitivity (563). For athletes, it is crucial that the body utilizes insulin with optimal efficiency throughout a physical change. A study investigating the impact of resveratrol on glucose revealed that resveratrol can enhance glucose regulation and insulin sensitivity in diabetic rats (564).

Consequently, many findings indicate that resveratrol may serve as a potent bioactive agent for athletes experiencing hyperglycemic swings and insulin resistance. Moreover, curcumin, a principal bioactive polyphenol found in the spice turmeric, exhibits notable antioxidant and anti-inflammatory activities. Due to its antioxidant properties, it effectively mitigates oxidative stress and promotes muscle regeneration through enhanced myofibrillar proliferation, hence decreasing muscle loss in an animal model of induced muscular atrophy (545). In human studies, curcumin supply resulted in a decrease in muscle damage and inflammatory biomarkers, with an approximate dosage of 150–1500 mg/day administered pre-, post-, and during exercise, potentially enhancing athletic performance and muscle repair by mitigating exercise-induced muscle injury and modulating the inflammatory reaction (565, 566).

Nevertheless, further investigation is required regarding the potential effects of curcumin supply on the molecular pathways that regulate muscle growth caused by resistance training. Furthermore, the advantages of curcumin are associated with its interaction with the gut bacteria. In animal studies, curcumin and resveratrol have anti-carcinogenic and anti-inflammatory properties on microbiota by altering the Firmicutes/Bacteroidetes ratio (567, 568). Curcumin enhances beneficial microbiome, involving lactobacilli, bifidobacteria, and butyrate*-*forming bacteria, while promoting intestinal barrier integrity through immunomodulatory effects (569, 570).

In a single-blind parallel-design clinical trial, Atan et al. (571) found that hardaliye ingestion increased total serum antioxidant capacity and decreased oxidative stress index and nitric oxide levels compared to the placebo group (571). The intake of hardaliye among young soccer players shows antioxidative properties (571). A distinct investigation comprising two sub-studies investigated the effects of sugar-polyphenol-rich diluted hazy apple juice on the intestinal barrier of ultra-marathon runners (572). The study findings indicated substantial impacts on indicators of intestinal inflammation and permeability in the serum of participants who took the test drink after exercise, yielding positive outcomes compared to those who ingested the placebo drink (572). Diluted apple juice was recognized for its rehydration properties post-exercise and can also positively influence the intestinal barrier and immunity following physical activities (572). Mengfan et al. (540) also indicate that polyphenolic substances from *Lonicera caerulea* may alleviate swimming fatigue at ambient and low temperatures. Moreover, the buildup of metabolites, energy metabolism, and the downregulation of inflammatory factor production were enhanced (540).

Tropospheric ozone, an element of urban air contamination, is generated via photochemical processes that involve nitrogen oxides, hydrocarbons, and volatile organic substances. Ozone exposure impacts the central nervous system, leading to neurological illnesses including Alzheimer’s and Parkinson’s diseases, cognitive deficits, and neuroinflammation (573). Both human and animal research demonstrate the neurotoxic consequences of ozone in this setting. These impacts encompass the diminution of dopaminergic neurons, the buildup of pathogenic proteins, and similar phenomena (573).

The hippocampus, a specific brain area, is susceptible to ozone exposure for multiple reasons. This area contains brain-derived neurotrophic factors and other elements important in neural growth, differentiation, memory, as well as learning (573). Research on brain-derived neurotrophic factors in humans and animals has demonstrated that short episodes of exercise enhance neuronal function, brain vascularization, and neuronal synthesis by increasing levels of derived neurotrophic factors, hence fostering improved mood and enhanced cognition (573). Nevertheless, exercising in contaminated air was demonstrated to suppress acute exercise-induced brain-derived neurotrophic factor release (573). Consequently, contact with contaminated air is believed to impede cognitive health and the repair of the central nervous system. A trial on high-intensity bikers showed that polyphenol supply elevated contaminated levels in those exercising in contaminated air (573).

**Figure 8:**
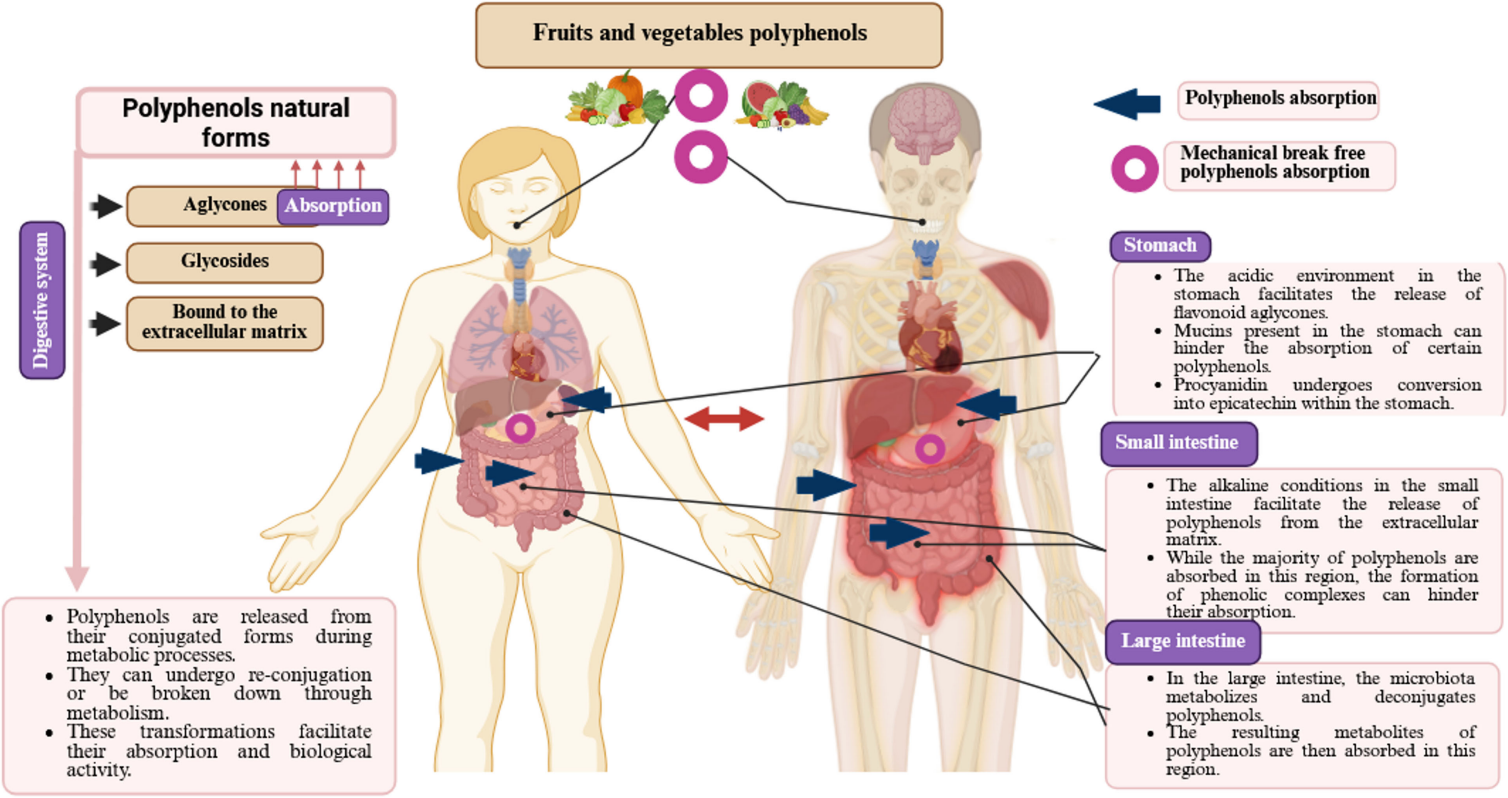
Polyphenol digestion and absorption in the human digestive system, depicting the natural forms of polyphenols (aglycones, glycosides, bound to extracellular matrix) found in fruits and vegetables, their mechanical breakdown and release in the stomach, absorption in the small intestine, microbial metabolism and metabolite absorption in the large intestine, and subsequent systemic circulation.

**Figure 8** illustrates the fate of dietary polyphenols within the human digestive system, beginning with their natural occurrence in fruits and vegetables as aglycones, glycosides, or bound forms. Mechanical processing in the stomach liberates these compounds, facilitating absorption in the small intestine, while unabsorbed fractions reach the large intestine for microbial metabolism, generating bioactive metabolites that subsequently enter systemic circulation. This metabolic journey has a direct impact on athletes’ health, as polyphenols exert antioxidant, anti-inflammatory, and regulatory effects on muscle metabolism, oxidative stress, and recovery. Thus, effective digestion and biotransformation of polyphenols underpins their capacity to enhance performance, endurance, and post-exercise repair.

### 6.4 Heart diseases and polyphenols

Globally, cardiovascular diseases are the leading cause of mortality, according to data from the World Health Organization (WHO). About 20 million fatalities annually, or 31% of all deaths, were attributed to cardiovascular diseases (574). Heart attacks and strokes account for around 85% of the fatalities listed above. Approximately 75% of worldwide cardiovascular disease-related mortality occurs in low- and middle-income nations (574). In accordance with the most current heart disease and stroke statistics published by the American Heart Association, it is estimated that more than 100 million people in the USA, which is equivalent to more than 50% of all people over the age of 18, have hypertension. Numerous health advantages and cardiovascular disease have been linked to polyphenols found in numerous dietary sources, for instance, apples, coffee, tea, and cocoa (575, 576).

Epidemiological research strongly suggests consuming polyphenols because it is unmistakably associated with a lower incidence of cardiovascular diseases (577, 578). Researchers now think that polyphenolic substances work at the molecular level to improve endothelial function and lower platelet aggregation because they can stop blood clots, reduce inflammation, and stop platelets from sticking together (401). Thus, polyphenolic substances are significant in the prevention and treatment of cardiovascular disease. According to some research, those who consume more flavonoids in their diets than those who consume the least have a 47% increased risk of cardiovascular disease (579).

Research has shown that consuming flavan-3-ol from various food sources may have positive effects on cardiometabolic outcomes and reduce the risk of diabetes and cardiovascular-related outcomes (such as blood pressure, cholesterol, and myocardial infarction). Flavan-3-ols, a well-recognized polyphenol, are found in significant concentrations in a number of frequently eaten foods, including tea, almonds, cocoa (chocolate), grapes, and legumes (580–582). Red and blue fruits and vegetables, including blueberries, raspberries, strawberries, bilberries, red grapes, and cherries, are rich sources of anthocyanins, a kind of flavonoid (583). Like other polyphenols, anthocyanins that are consumed through diet are metabolized by the microbiome and the host to create active metabolites that have anti-inflammatory properties, improve vascular outcomes, reduce the risk of myocardial infarction in both men and women, and have additional positive effects on cardiovascular risk factors (583, 584).

Stilbene is mainly found in berries, red wine, and grapes. In addition to its antioxidant and anti-inflammatory properties, it also stimulates sirtuins, which slow down the aging process (407). Resveratrol supplements are said to significantly reduce fasting blood sugar, total cholesterol, C-reactive protein, and both systolic and diastolic blood pressure (585). Apple flavonol quercetin has been shown to lower systolic blood pressure, improve endothelial function, and lessen the risk of cardiovascular disease (585–587).

### 6.5 Polyphenols and Alzheimer’s disease

Alzheimer’s disease is a catastrophic neurodegenerative condition that affects elderly people worldwide (588). Damage to neuron structure and function is the primary cause, which ultimately results in the death of nerve cells in the human brain (588). WHO has disclosed that over 50 million people globally suffer from dementias, including Alzheimer’s disease, and that it is expected to rise to over 152 million by the year 2050. Approximately 60% of dementia patients globally originate from low- or middle-income nations (589).

Alzheimer’s disease is thought to be at risk due to both genetic and environmental factors (590). Free radicals are very reactive chemical groups that arise from both physiological and pathological processes. They have an odd number of electrons. At normal concentrations, ROS participate in many cellular and signaling pathways, including phagocytosis, enzyme activation, and cell cycle control (591). However, excessive ROS generation may lead to harmful consequences, such as damage to proteins, lipids, and DNA (591). Cell damage may result from an imbalance in the status of oxidants and antioxidants. It has been proposed that oxidative damage, a consequence of ROS, plays a role in the pathogenesis—the formation, process, and advancement of neurodegenerative diseases and disorders, cancer, diabetes, and aging (592).

Extensive scholarly research has shown that nitric oxide, hydrogen peroxide, hydroxyl radicals, and superoxide anion are essential components of oxidative stress, which ultimately results in Alzheimer’s disease (593). However, the defensive systems known as enzymatic and non-enzymatic antioxidants eliminate ROS. Polyphenolic chemicals have antioxidant properties and are primarily involved in neuroprotection. Pomegranate juice, dates, and figs are all high in polyphenols and should be added to the diet to help with behavioral problems and brain damage by keeping the balance between oxidants and antioxidants in transgenic APPs w/T g 2576 animals (594). Additionally, researchers found that extract from walnuts, whose polyphenols are the most effective among all nuts, has a remarkable ability to shield PC12 cells from oxidative stress caused by amyloid beta peptide (594).

### 6.6 Polyphenols’ anti-cariogenic properties

Tooth decay, or dental caries, affects 60–90% of children and most adults worldwide and is one of the most common and serious oral health issues (595). Teeth, oral flora, and nutritional factors all have an impact on dental caries disease. Dental plaque absorbs dietary carbohydrates like sucrose or sugars, which bacteria (found in dental plaque on the outside of teeth) then convert into organic acids like lactic acid (595). Demineralization, or the net loss of mineral structure on the tooth’s surface, is the result of the acid produced gradually removing calcium and phosphate from the tooth’s surface (595).

Polyphenols, which are present in tea, coffee, red grape seeds, and cocoa, have antibacterial properties that may help prevent cariogenic processes. They may slow down the growth of bacteria, protect the tooth surface, and inhibit the activity of enzymes like glucosyltransferase and amylase. Flavonoids are also effective anti-cariogenic compounds (596).

Two categories can delineate the anti-cariogenic properties of phenolic compounds: (I) plant extracts rich in polyphenols without recognized constituents; and (II) antibacterial polyphenolic agents. It has been shown that extracts derived from unfermented cocoa, green tea, and red grape seeds that include a high polyphenol concentration are effective against *Streptococcus mutants* and periodontal disorders. A flavonoid called quercetin-3-O-α-L-arabinose-pyranoside (guaijaverin) stops the growth of *S. mutants*, which is likely an anti-plaque effect (597, 598).

## 7 Possible negative impact of polyphenols

People use polyphenols, an essential component of plant-derived food with numerous health benefits, to prevent and cure multiple diseases (599). Tragically, like any chemical substance, polyphenols may be harmful depending on dosage, circumstances, and environmental interactions. One of the important negative consequences of polyphenols is their ability to block iron uptake in the human body (599). Despite being a trace element that is necessary for human survival, iron deficiency is a widespread ailment that affects people all over the globe (599). Polyphenols have the capacity to bind to transition metal ions such as iron and copper. This stops free radicals from being produced by the Fenton and Haber-Weiss reactions (600). In addition to the polyphenolic compound’s structure, the pH or ion form (Fe^2+^ and Fe^3+^) influences both binding strength and total ion concentration. Anemia occurs when an individual consumes a diet rich in polyphenols or takes supplements containing these compounds (600). Polyphenols bind to iron in the gastrointestinal tract, inhibiting its absorption. Additionally, they may influence the regulation of iron homeostasis (599, 600).

Flavonoids can form complexes of proteins by both nonspecific mechanisms such as hydrogen bonding and hydrophobic effects, as well as with covalent bond formation. Polyphenols form complexes with proteins, which may be either soluble or insoluble. These complexes alter the structure, isoelectric point, hydrophobicity, solubility, and susceptibility of the proteins to enzymes (601). Polyphenols may have detrimental effects on the digestive system’s function by impacting the composition of the intestinal flora and inhibiting digestive enzymes (602).

## 8 Conclusions and future perspective

Polyphenols, a diverse class of natural compounds, exhibit a wide range of biological activities largely determined by the number and position of their hydroxyl groups. Abundant in herbs and prevalent in traditional Asian and Mediterranean diets, these compounds have attracted significant scientific interest for their potential health benefits. Despite extensive research highlighting their neuroprotective, antioxidant, anti-inflammatory, antibacterial, dermatological, antitumor, and antidiabetic properties, the underlying mechanisms remain only partially understood.

A key limitation in translating these benefits into clinical practice lies in the low bioavailability of polyphenols. The practical improvement of daily polyphenol intake can be achieved through several strategies. Consuming a diverse range of polyphenol-rich foods, such as fruits, vegetables, whole grains, nuts, tea, coffee, and certain herbs across meals throughout the day, ensures both variety and a steady supply of these bioactive compounds. Choosing minimally processed foods and combining different sources may enhance overall intake and synergistic effects. Additionally, adopting preparation methods that preserve polyphenol content, such as steaming instead of boiling, and consuming polyphenol-rich foods with healthy fats may improve absorption.

Additionally, polyphenols that benefit most from nano-delivery systems are those with inherently poor water solubility, instability in the gastrointestinal tract, or rapid metabolism, factors that limit their absorption and therapeutic potential. Notably, curcumin, quercetin, resveratrol, tea polyphenols such as EGCG, and catechins have shown significant improvements in bioavailability, biological activity, and stability when encapsulated in nanoscale carriers. Nano-delivery approaches also enhance the performance of lignans and tannic acid, as well as complex polyphenolic extracts from sources like grape seed and propolis. By protecting these compounds from degradation and promoting controlled, targeted release, nano-delivery systems make these polyphenols more effective for use in health and disease management applications.

Many are rapidly metabolized or degraded before reaching their target tissues, reducing their therapeutic potential. To address this, advanced drug delivery systems such as liposomes and nanocarriers have been widely investigated. However, a universally effective delivery method applicable across different polyphenol classes is yet to be established, highlighting the need for further targeted research. Polyphenols continue to be an important focus in nutritional science, with growing evidence supporting their role in health maintenance across various populations, including athletes. Yet, current literature lacks consensus on optimal intake levels, and comprehensive studies encompassing all polyphenol subclasses are still limited.

Looking forward, enhancing the bioavailability and targeted delivery of polyphenols could open new avenues in drug development for metabolic disorders, dermatological applications, and the formation of functional foods aimed at improving physical performance and overall well-being. Bridging current knowledge gaps and integrating polyphenols into daily dietary practices may contribute significantly to promoting healthier lifestyles and improving performance outcomes, particularly among future generations.
